# Improved thermal behavior of polypropylene polymer loaded with bulk and nanoscale Bi_2_O_3_/CuO composite

**DOI:** 10.1038/s41598-025-26980-4

**Published:** 2025-12-01

**Authors:** Mohamed S. Badawi, M. Y. El Sayed, Mona Shebly, Amro Obeid, K. Habanjar, R. Awad

**Affiliations:** 1 Faculty of Science, Department of Physics, Alexandria University, Alexandria, Egypt; 2 Faculty of Science, Alamein International University, Alamein City, Egypt; 3 Faculty of Health Sciences, Public Health Department, Modern University for Business and Science, Beirut, Lebanon; 4 Faculty of Science, Department of Physics, Beirut Arab University, Beirut, Lebanon; 5 Lebanese Atomic Energy Commission, National Council for Scientific Research, Beirut, Lebanon; 6 Faculty of Computer Science and Artificial Intelligence, Department of Basic Sciences, Pharos University in Alexandria, Alexandria, Egypt

**Keywords:** Nanocomposite, Nanopolymer composite, Thermal analysis, Degree of crystallinity, Materials science, Nanoscience and technology

## Abstract

This study investigates the structural, vibrational, morphological, and optical properties of bulk and nanoscale Bi_2_O_3_ and CuO oxides and their composite, which were prepared via high-speed ball milling at different times (20, 40, 60, and 80 min). According to X-ray diffraction analysis, Bi_2_O_3_ and CuO preserved their monoclinic crystal structures. However, the increase in milling time resulted in peak broadening, smaller crystallite sizes, and higher microstrain. FTIR revealed consistent Bi–O and Cu–O vibrational modes, with slight shifts to higher wavenumbers as crystallite sizes decreased. PL investigations revealed a significant quenching effect with prolonged milling, which was attributed to an increase in oxygen vacancies and structural defects, leading to enhanced related emissions. The EDX verified the elemental compositions, and SEM micrographs demonstrated how increasing milling time transformed the bulk agglomerates into finer and more uniform nanoparticles. In both bulk and nanocomposites, the 70 wt.% Bi_2_O_3_ and 30 wt.% CuO combination displayed encouraging optical characteristics, indicating potential for usage in optoelectronic devices. The effect of bulk and nanofillers on the thermal and structural properties of polypropylene (PP) composites is also studied in this research. According to thermogravimetric analysis studies, the resistance to heat degradation significantly improves with an increase in filler content. The highest bulk (B20) and nanofiller (N20) composites had commencing breakdown temperatures (T_5%_) of 381 °C and 387 °C, respectively, compared to 324 °C for pure PP. Due to improved filler dispersion and interfacial contact, nanocomposites exhibited advanced thermal barrier performance with higher activation energies and a more consistent and regulated degradation profile. Particularly in nanocomposites, DSC data showed a little change in the melting onset and peak temperatures along with an increase in the degree of crystallinity and enthalpy of fusion. The outcomes demonstrate that although both types of fillers enhance thermal behavior, nanofillers provide better improvements due to their greater surface area and stronger interfacial effects. Incorporating nanoscale fillers into polymer nanocomposites is crucial for improving thermal stability, heat resistance, and crystallinity because they facilitate efficient heat transfer and function as powerful nucleating agents inside the polymer matrix.

## Introduction

The thermoplastic polymer polypropylene (PP) possesses a unique combination of characteristics that make it suitable for a wide range of industrial applications. It is ideal for lightweight and long-lasting products due to its exceptional mechanical strength, low density, and exceptional chemical resistance^1^. Because it is semi-crystalline and has a relatively high melting point, often between 160 and 170°C, it can withstand significant heat stress^2^. Polypropylene is used extensively in textiles, medical devices, automotive parts, and packaging. It is also resistant to a broad variety of acids, bases, and organic solvents^3^. Additionally, its recyclability and ease of processing via techniques like extrusion and injection molding make it an economical and environmentally beneficial material^4^. The addition of metal oxide nanoparticles to PP has been investigated recently in an effort to improve its functional characteristics. Gamma-ray attenuation has been improved when bismuth oxide (Bi_2_O_3_) nanoparticles are added to PP, indicating their potential for radiation shielding applications. In comparison to microscale particles, the study found that nanoscale Bi_2_O_3_ offered superior morphological dispersion and shielding efficacy without sacrificing the integrity of the polymer^5^. In a similar vein, adding copper oxide (CuO) nanoparticles to PP fibers produced significant antibacterial effects without compromising structural integrity. The composite fibers demonstrated good antibacterial efficacy, indicating their applicability for protective and medical textile applications, especially those with specified cross-sectional geometries^6^. These results validate the use of Bi_2_O_3_ and CuO as functional fillers in PP to provide enhanced bioactive and protective properties. However, its applications are sometimes subject to many limitations in thermal and structural stability. To address these issues, researchers have explored the incorporation of inorganic fillers into the polymer matrix, particularly CuO and Bi_2_O_3_ nanoparticles, due to their favorable physicochemical properties^7,8^.

CuO and Bi_2_O_3_ nanoparticles have garnered a lot of attention due to their unique physicochemical and functional features, which suit them for a range of industrial and functional uses. CuO nanoparticles exhibit excellent electrical conductivity, thermal stability, and strong antibacterial activity due to their huge surface area and ability to generate reactive oxygen species^9^. CuO nanoparticles have been extensively utilized in gas sensing, catalysis, supercapacitors, and lithium-ion battery production^7^. The high refractive index, broad bandgap, and remarkable photoconductivity of Bi_2_O_3_ nanoparticles, on the other hand, make them very useful in photocatalytic and optoelectronic applications^10^. Bi_2_O_3_ is also a promising material for environmental and medical applications due to its excellent antibacterial properties and low toxicity^11^. The nanoscale dimension of these oxides significantly increases their surface reactivity and functional flexibility as compared to their bulk counterparts.

Therefore, it is intriguing to include nanoparticles in polymers to enable their application under challenging conditions^12^. The effect of adding metal oxides, particularly CuO and Bi_2_O_3_ nanoparticles, in improving the characteristics of the produced composites was the subject of numerous investigations. Polymer nanocomposites based on PP/Cu-CuO were created by Ramazanov et al.^13^ by combining the techniques of hot pressing and ex-situ casting solutions. They discovered that the polarizability of these materials was improved and that the dielectric permittivity of nanocomposites increased. Bagheri et al.^14^ examined and compared the mechanical, radiation-resistant, and physical characteristics of several polymer composites using 60-weight percent Bi_2_O_3_ as reinforcement. The computed gamma-ray attenuation coefficients show a remarkable rise in the composite’s attenuation power, and they are in reasonably good agreement with the experimental data. Additionally, they demonstrated that the materials with the highest and lowest elastic modulus and strength, respectively, are epoxy, PP, HDPE, LDPE, and PVA.

Thus, the physical, mechanical, thermal, and functional characteristics of the resultant nanocomposites are greatly improved by the addition of nanoparticles to polymer matrices. Nanoparticles such as metal oxides, carbon-based compounds, and clays can improve the tensile strength, stiffness, and barrier properties of polymers by fortifying the polymer structure at the nanoscale^15^. These enhancements are mostly due to the broad surface area and high aspect ratio of nanoparticles, which promote strong interfacial contact with the polymer chains^16^. Additionally, depending on their nature and distribution within the polymer, nanoparticles might impart new capabilities like UV protection, electrical conductivity, flame retardancy, and antibacterial activity^17^. Overall, adding nanoparticles to polymers is a promising way to customize their performance for advanced technical and medicinal uses.

In this work, CuO and Bi_2_O_3_ nanoparticles are synthesized and integrated into a PP matrix to form a novel class of nanocomposites. Despite having beneficial functional characteristics, the utilization of CuO and Bi_2_O_3_ nanoparticles as co-fillers in polymer nanocomposites is a relatively unexplored prospective use with promising material enhancement implications. To balance and complement the unique functional characteristics of both metal oxides, a 70% Bi_2_O_3_ and 30% CuO ratio was chosen for the nanocomposite. Bi_2_O_3_ is the perfect dominant phase for structural and protective applications because of its high atomic number and density. On the other hand, CuO adds beneficial advantages such as enhanced thermal conductivity, electrical conductivity, and antibacterial activity^5^. The composite preserves overall material integrity by reducing the likelihood of nanoparticle aggregation and ensuring improved dispersion within the matrix by keeping CuO at 30%. The 70:30 composition is useful for multifunctional polymer-based nanocomposites since it also reflects the ideal ratios documented in the literature, where CuO improves secondary performance aspects and Bi_2_O_3_ acts as the principal functional phase. The primary objective of this study is to investigate the structural and thermal behavior of PP modified with CuO/Bi_2_O_3_ nanocomposite to enhance its performance under thermal stress and increase its range of applications. This paper provides insights into the effects of hybrid metal oxide fillers on the crystallinity, morphology, and thermal stability of polypropylene, thereby contributing to the expanding field of multifunctional polymer nanocomposites. The novelty of this work does not only stem from the specific 70/30 Bi_2_O_3_-CuO ratio but from the comprehensive exploration of how mechanical milling modulates the structural, optical, and thermal properties of these oxides and their corresponding PP composites. The study establishes clear links between particle size reduction, defect generation, photoluminescence behavior, and polymer–filler interfacial enhancement, providing new insight into structure and property relationships in polymer/oxide hybrid systems.

This study is innovative because it synthesizes and compares bulk and nanoscale CuO and Bi_2_O_3_ nanoparticles and integrates them into a PP matrix. First, bulk CuO and Bi_2_O_3_ nanoparticles were produced, and then they were mechanically milled for 20, 40, 60, and 80 min to reduce their size and produce nanoscale particles. The nanoparticles that underwent a 60-min milling process were chosen for composite production because of their optimal structural properties. The bulk CuO and Bi_2_O_3_ powders (bulk composite) and the nanoparticles milled for 60 min (nanocomposite) were used to produce the two different kinds of metal oxide blends. The effects of filler size on the properties of the polymer were next investigated by incorporating these composites into the PP matrix at varying concentrations (0, 5, 10, 15, and 20 wt.%). To shed light on how particle size and filler concentration affect polymer performance, the study intends to investigate and contrast the structural and thermal behavior of PP-based composites reinforced with bulk and nanoscale CuO-Bi_2_O_3_ fillers. Utilizing methods like X-ray diffraction (XRD), Fourier-transform infrared spectroscopy (FTIR), scanning and transmission electron microscopy (SEM–EDX, TEM), and photoluminescence (PL) analysis, a thorough investigation is conducted to assess the structural and optical characteristics of the synthesized nanoparticles and their composite. Furthermore, using XRD, thermogravimetric analysis (TGA), and differential scanning calorimetry (DSC), the effect of adding the CuO-Bi_2_O_3_ bulk composite and nanocomposite on the thermal and structural behavior of PP is investigated. This study aims to shed light on the preparation of innovative polymer-based nanocomposites with enhanced structural and thermal stability.

## Experimental work

### Materials

Table 1 lists the initial materials used in this work.

**Table 1:** Materials purity and supplier information.

Material	Company	Purity	Density
Bismuth oxide (Bi_2_O_3_)	Sigma-Aldrich	98%	8.9 g/cm^3^
Copper oxide (CuO)	Sigma-Aldrich	98%	6.31 g/cm^3^
Polypropylene (PP)	Egyptian Propylene and Polypropylene Company	99%	0.9 g/cm^3^

### Bi_2_O_3_ and CuO nanoparticles preparation

To start the preparation method of Bi_2_O_3_ or CuO, a mechanical method chosen by a high-energy planetary ball mill (Retsch, PM 100) was utilized in a dry state for different times (20, 40, 60, and 80 min) with the following conditions: the ball-to-powder weight ratio was 10:1, and the milling speed was set at 400 rpm. The milling was stopped for 5 min every 20 min to cool down the system. The bulk and ball-milled Bi_2_O_3_ samples milled for 20, 40, 60, and 80 min are designated as Bi₂O₃-Bulk, Bi_2_O_3_-20, Bi_2_O_3_-40, Bi_2_O_3_-60, and Bi_2_O_3_-80, respectively. Similarly, for CuO, the bulk and milled samples are labeled as CuO-Bulk, CuO-20, CuO-40, CuO-60, and CuO-80.

### Bi_2_O_3_ /CuO bulk and nanoparticle composites preparations

To ensure homogeneity for bulk and nano-mixtures, commercial or obtained nano Bi_2_O_3_ (70%) and CuO (30%) were used. They were dispersed in pure ethanol and stirred for around 60 min. The solvent was evaporated at 60 °C during stirring.

### PP/(bulk and nano composites) synthesis

Various weight fractions of bulk and nano (Bi_2_O_3_-CuO) filler (5, 10, 15, and 20 wt.%) were produced through the compression molding process. The required amount of PP was measured and melted in a two-roll mill mixer (XK400, Shandong, China) at 170 °C for 10 min with a rotation speed of 50 rev/min. Following this, the mixture was blended for an additional 15 min, during which either bulk composite or nanocomposite was incrementally added to the blend. The resultant mixes were milled for 10 min to ensure the filler was dispersed uniformly throughout the polymer matrix. Subsequently, the samples were crushed and positioned between Teflon layers to create a smooth sheet inside a rectangular stainless steel mold that measured 25 × 25 × 0.3 cm. In addition to molding, tooling, and heating capabilities, the sheet was then hot pressed at 20 MPa utilizing hydraulic compression molding (Shanghai, China) at 170 °C for 15 min. After being sintered, the sample was compressed and cooled at a rate of 20 °C per minute. The produced sheet was cut into 3 × 3 mm square sections to create standard test specimens appropriate for Vickers microhardness testing. In this manuscript, pure PP is referred to as B0. Bulk fillers were incorporated into the PP matrix at concentrations of 5, 10, 15, and 20 wt. %, and the resulting composites were designated as B5, B10, B15, and B20, respectively. Similarly, nanofillers were added at the same concentrations, and the corresponding samples were labeled as N5, N10, N15, and N20.

## Characterization techniques

A Bruker D8 Advance instrument with Cu-Kα radiation (λ = 1.5406 Å) was used to perform XRD on the produced samples to analyze their structure. By using CIF files from the Crystallography Open Database, the diffractometer captured the diffraction patterns, which were then refined using the Rietveld method and Material Analysis Using Diffraction (MAUD) software. The Nicolet iS5 spectrometer was used to record FTIR spectra in the wavenumber range of 4000–400 cm^-1^. Scanning electron microscopy (SEM) and energy-dispersive X-ray spectroscopy (EDX) were used to examine the morphology and elemental makeup of the produced samples. These measurements were conducted with a JEOL JCM-6000PLUS with an EX-54450U1S61 detector, focusing on different regions of the samples at an operating voltage of 20 keV. A Transmission Electron Microscope TEM (JOEL, JEM-2100) was employed at an accelerating voltage of 20 kV to examine the microstructure, agglomeration, and particle size distribution. Particle sizes were measured from TEM images using ImageJ software. Additionally, PL spectra were attained at room temperature using a fluorescence spectrometer (FP8300) by applying an excitation wavelength of 330 nm. The thermal degradation analysis of the synthesized composites was conducted using a (TGA-DTA/DSC SETARAM- Labsys). The chosen operational parameters included a heating range from 25 to 600 °C and a heating rate of 10 °C/min in a pure nitrogen environment. The sample mass ranged from 10 to 15 mg and was placed in alumina (Al_2_O_3_) crucibles.

## Results and discussion

### Characterization of bulk and nanoscale Bi_2_O_3_–CuO oxides

#### X-ray diffraction (XRD) analysis

i. Bulk and milled Bi_2_O_3_ and CuO nanoparticles

**Fig. 1:**
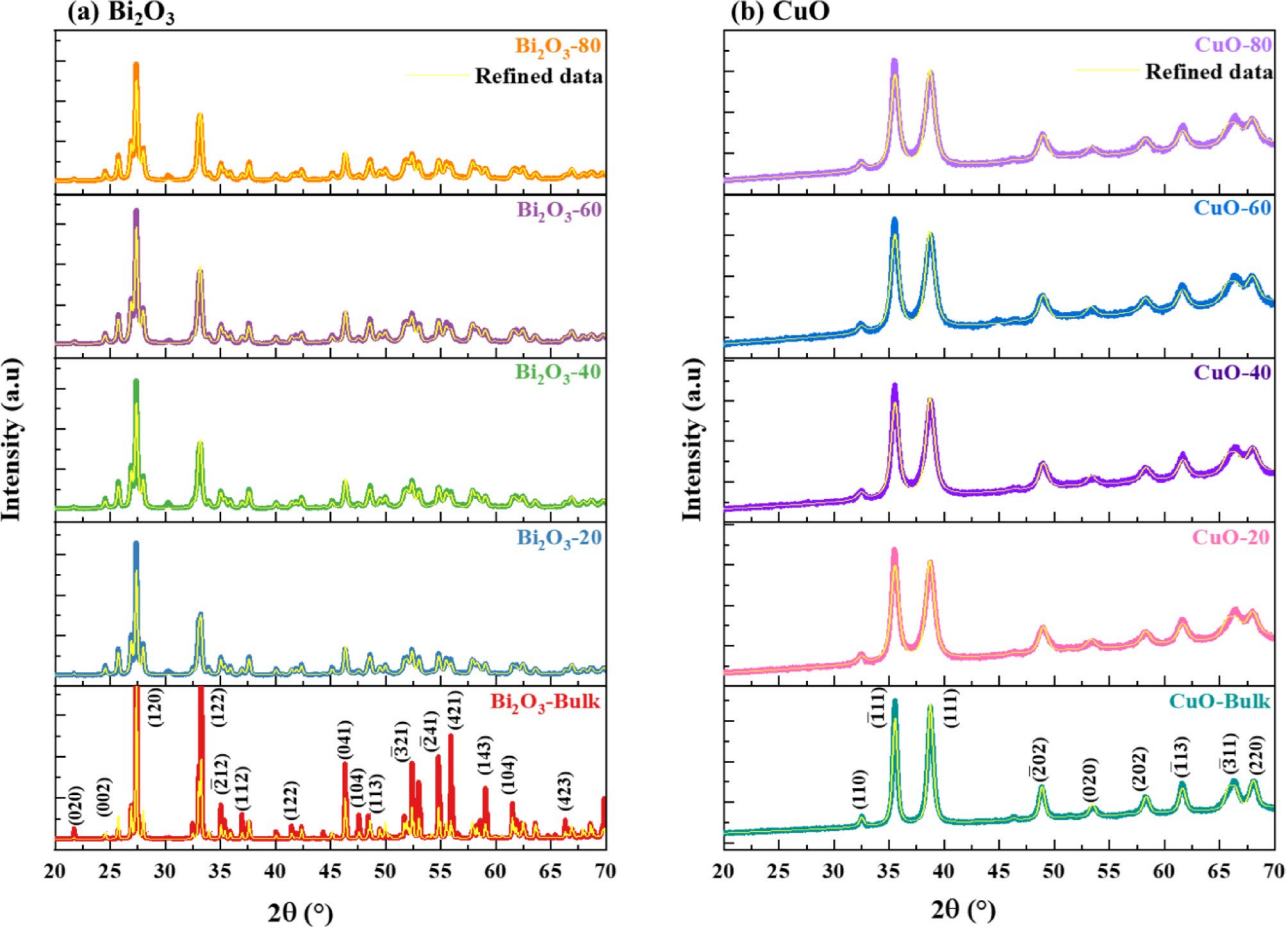
XRD patterns of bulk and milled (**a**) Bi_2_O_3_ and (**b**) CuO nanoparticles.

**Fig. 2:**
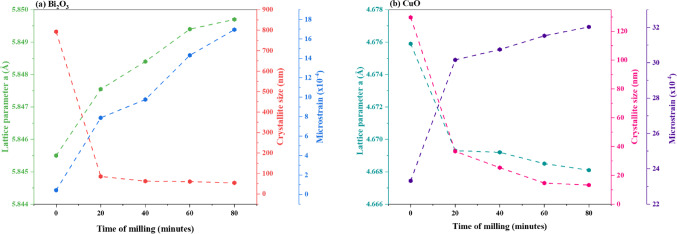
The variation of crystallite size, lattice parameters, and microstrain of bulk and milled Bi_2_O_3_ and CuO.

Figure 1(a) displays the XRD patterns of bulk and milled Bi_2_O_3_ at various milling times (20, 40, 60, and 80 min). The refined diffraction peaks across all samples of Bi_2_O_3_ displayed similar positions with no tangible shift, confirming that no significant phase transformation occurred between the samples. The main diffraction peaks at 2θ values of 27.35°, 33.28°, 35.08°, 36.93°, 46.32°, 52.36°, 54.80°, 55.89°, and 59.07°, correspond to planes (120), (122), ($\overline{2 }$ 12), (112), (041), ($\overline{3 }$ 21), ($\overline{2 }$ 41), (421), and (143), indicating the monoclinic structure of Bi_2_O_3_^18,19^. The strength and sharpness of the bulk Bi_2_O_3_ characteristic peak intensities indicate its high crystallinity. On the other hand, as the milling duration rose, the XRD patterns of milled Bi2O3 revealed peak broadening and a modest decrease in intensity; the sample that was milled for 80 min displayed the lowest and broadest peak. This supports the observations in Fig. 2, where an increase in microstrain and a reduction in crystallite size were noted with increasing milling time of Bi_2_O_3_^20^. Additionally, the lattice parameter ($a$), which denotes the refined unit cell dimension obtained from XRD data and reflects the structural strain and distortion induced by the mechanical milling process, increased from 5.8455 to 5.8497 Å as the milling time extended to 80 min, as estimated using MAUD software. This increase in lattice parameters is primarily attributed to the lattice strain and defects induced by the mechanical milling process.

Figure 1(b) illustrates the XRD patterns of bulk and milled CuO subjected to the similar milling times used for Bi_2_O_3_ (20, 40, 60, and 80 min); likewise, the refined diffraction peaks of CuO samples showed nearly the same position with no remarkable shift, which indicated the monoclinic structure of CuO. The main diffraction peaks at 2θ values of 35.43°, 38.71°, 48.76°, 61.54°, 66.26°, and 68.08° correspond to planes ($\overline{1 }$ 11), (111), ($\overline{2 }$ 02), ($\overline{1 }$ 13), ($\overline{3 }$ 11) and (220)^21^. Similarly, it can be observed a broadening and slight decrease in the peak intensity of CuO samples as the milling time increases. The lowest and widest peak intensity was also observed for the CuO sample milled at a time of 80 min, thus confirming the abundance of microstrain and the smallest crystallite size, as shown in Fig. 2^22^. After milling, the crystallite size decreases, allowing atoms to reorganize into a denser packing configuration and causing a minor lattice contraction, which is responsible for the drop in the lattice parameter ($a$) of CuO (Fig. 2).

For the various milling times (20, 40, 60, and 80 min), the average crystallite sizes of the milled Bi_2_O_3_ and CuO samples were calculated using Scherrer’s Eq. (1) and were estimated to be 85 nm, 62 nm, 60 nm, and 54 nm for the milled Bi_2_O_3_ samples and 37 nm, 25 nm, 15 nm, and 13 nm for the milled CuO samples.

1 $$D = \frac{0.9\lambda }{{\beta \cos \theta }}$$

where *D* is the mean crystallite size, λ is the wavelength of Cu-Kα, ($\lambda$ = 1.5405 Å), *β* is the full width at half maximum intensity of the peak (FWHM) in radians, and 2*θ* is Bragg’s diffraction angle.

The obtained results of the crystallite size using the mechanical milling process were comparable to other studies using different methods, where Bi_2_O_3_ nanoparticles prepared by a sol–gel method had a size of 37.88 nm^23^ while others had a size range between 64.02 and 17.03 nm^24^. The CuO nanoparticles prepared by a wet chemical precipitation method had a size of 34 nm^25^, and others had a size range of 23.09 − 21.33 nm, prepared by the co-precipitation method^26^.

ii. Bi_2_O_3_-CuO nanocomposite

**Fig. 3:**
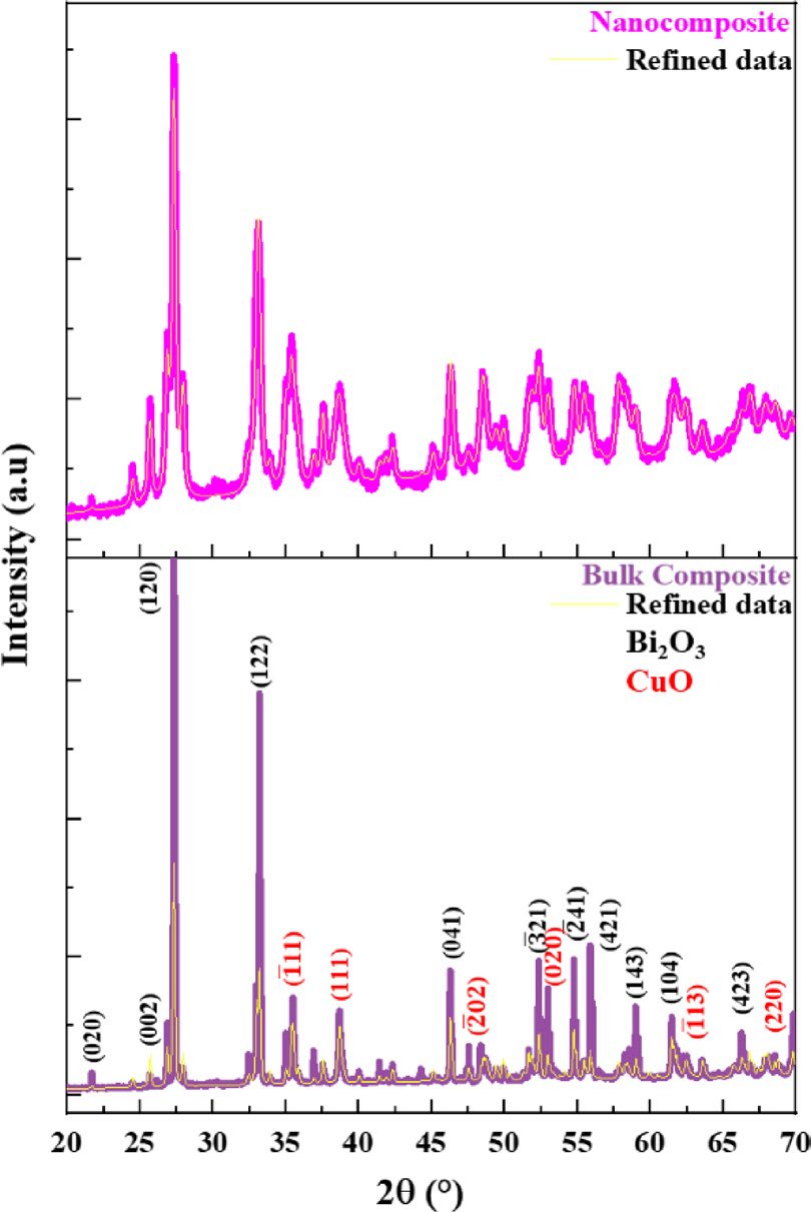
XRD patterns of (70 wt% Bi_2_O_3_/30 wt% CuO) bulk and nanocomposite.

As shown in Fig. 3, the two weight fractions, 70% of Bi_2_O_3_ and 30% of CuO, were chosen to give remarkable blend fillers for both bulk and nano (Bi_2_O_3_/CuO) composites. The presence of the main diffraction peaks corresponding to Bi_2_O_3_ and CuO phases confirms the successful formation of the Bi_2_O_3_-CuO nanocomposite. The diffraction peaks in bulk and nano (Bi_2_O_3_-CuO) fillers exhibited almost the same position with no significant shift; Any minor apparent variations observed do not represent actual structural shifts but are within instrumental tolerance and result primarily from peak broadening caused by nanoscale strain, and the reduction in overall crystallinity. A detailed quantitative crystallinity assessment of PP-based composites and its correlation with mechanical performance are reported separately in a forthcoming study.

#### Fourier-Transform Infrared (FTIR) Spectroscopy

i. Bulk and milled Bi_2_O_3_ and CuO nanoparticles

**Fig. 4:**
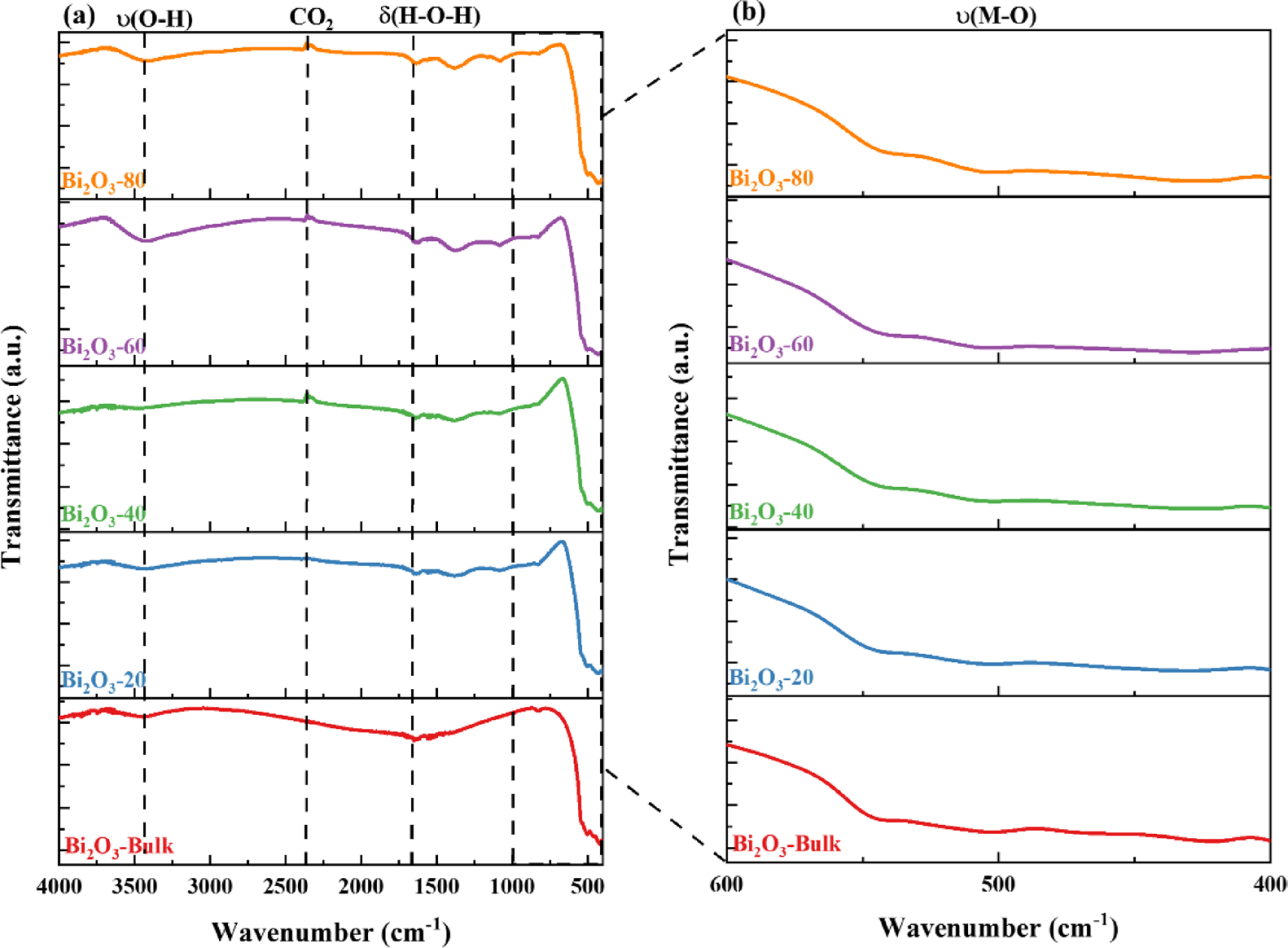
FTIR spectra of bulk and milled Bi_2_O_3_ samples.

**Fig. 5:**
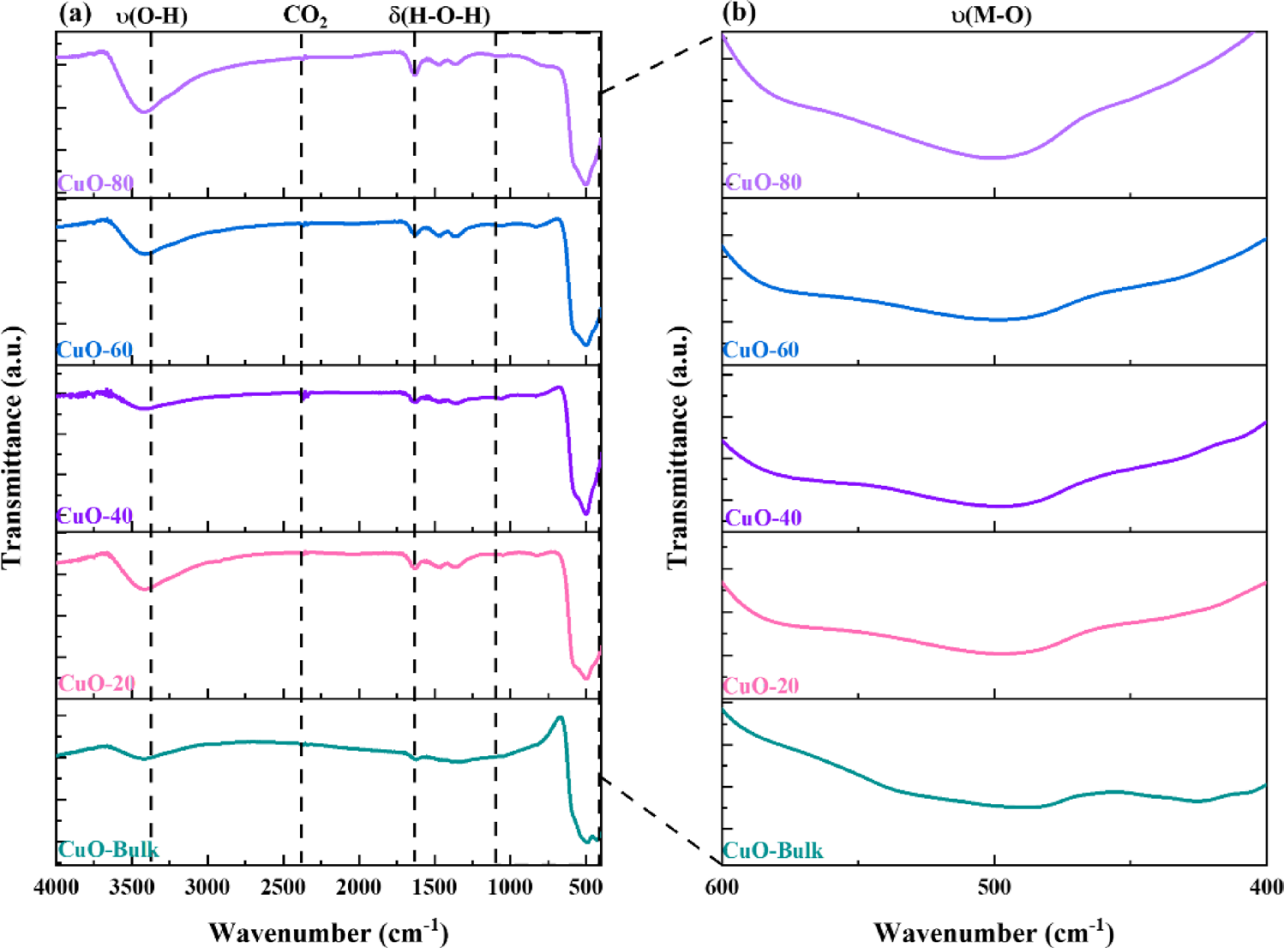
FTIR spectra of bulk and milled CuO samples.

The FTIR spectra of bulk and milled Bi_2_O_3_ and CuO at different milling times (20, 40, 60, and 80 min) are shown in Figs. 4 and 5. The main Bi_2_O_3_ and CuO vibrational modes were observed in the region 4000–400 cm^-1^. For the bulk Bi_2_O_3_ sample, two characteristic peaks at 502.24 cm^-1^ and 429.04 cm^-1^ were detected and referred to as (Bi-O) stretching vibrations^27,28^. Whereas, these peaks were shifted to (504.54–505.49 cm^-1^) and (429.46–431.95 cm^-1^) as the milling time of Bi_2_O_3_ increased, respectively. The milling causes particle size reduction, leading to lattice strain, shortening the bond lengths, and leading to higher vibrational stretching, which is confirmed by the shifting to higher wavenumbers^29,30^. Similarly, the bulk CuO sample shows a characteristic peak at 487.67 cm^-1^, and referred to as (Cu–O) stretching vibrations. The characteristic peak of CuO is shifted to a higher wavelength (497.49- 500.03 cm^-1^) as the milling time of CuO increases from 20 to 80 min, respectively^31,32^. Furthermore, because water from the environment is physically adsorbed, the peaks in the 3445.24 cm^-1^ and 1636.78 cm^-1^ range correspond to the ν(O–H) stretching vibrations and δ(H–O-H) bending vibrations of the adsorbed water’s hydroxyl group, which are present in all spectra. Additionally, a minuscule signal seen within the 2368.15 cm^-1^ range indicated the presence of carbon dioxide in the atmosphere^33^.

The characteristic bands observed for milled Bi_2_O_3_ and CuO are consistent with those reported in the literature, including Bi_2_O_3_ synthesized via chemical precipitation^34^ and co-pyrolysis methods^35^ Bi_2_O_3_ and CuO were synthesized by the sol–gel and co-precipitation methods, respectively^36^, as well as CuO prepared using a simple precipitation method^37^.

ii. Bi_2_O_3_-CuO nanocomposite

**Fig. 6:**
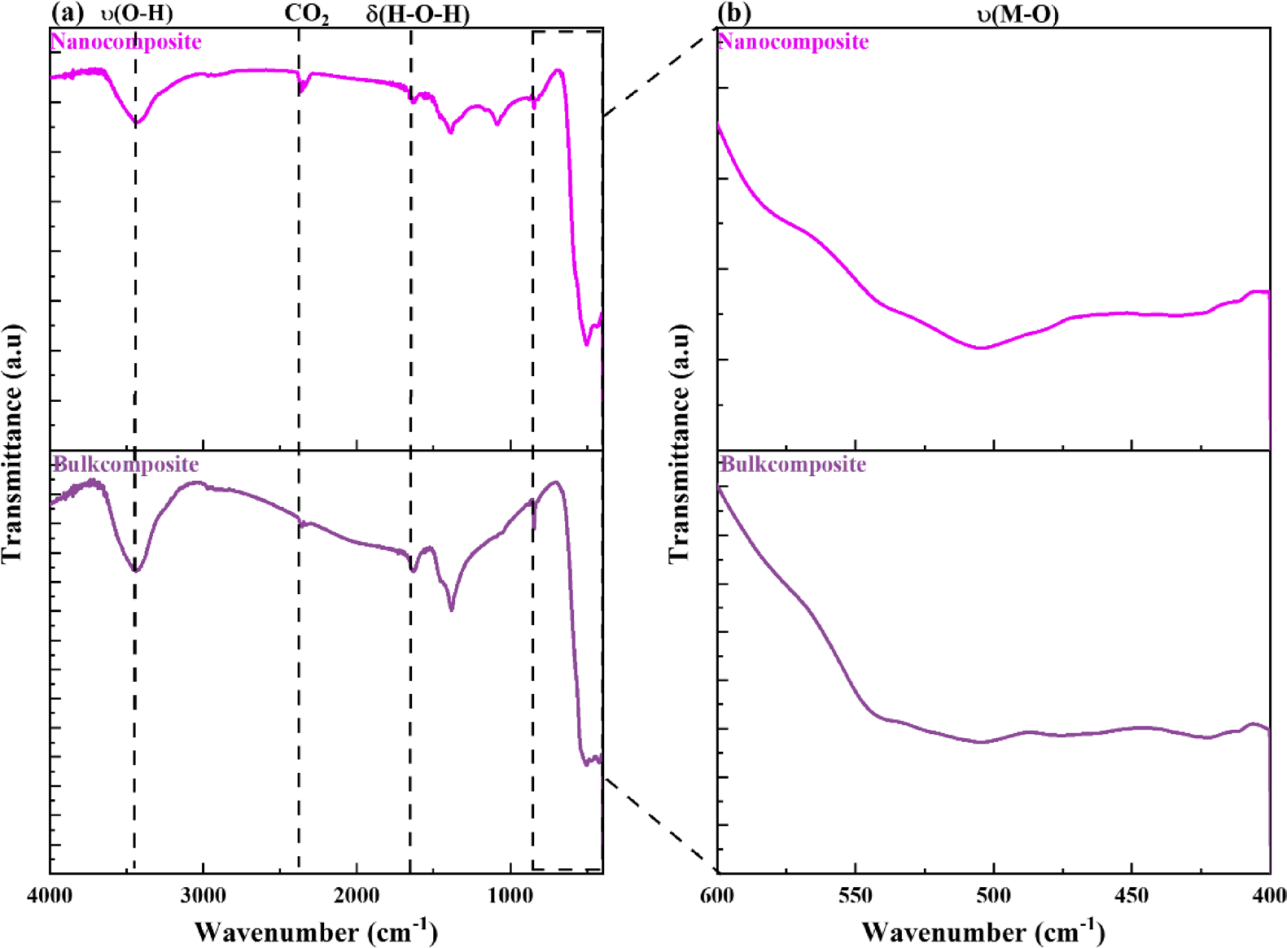
(**a**) FTIR spectra of (70 wt% Bi_2_O_3_/30 wt% CuO) bulk and nanocomposites, and (**b**) shows the zoom-in of the fingerprint region.

A complementary FTIR spectra for the two weight fractions 70% of Bi_2_O_3_ and 30% of CuO were chosen to give remarkable blend fillers for both bulk and nano (Bi_2_O_3_/CuO) composites as shown in Fig. 6. The characteristic peaks observed in the range between 504.94 and 422.16 cm^-1^ could be assigned to the stretching vibrations of both bulk and nano (Bi_2_O_3_/CuO)^27,29,32^. In addition, the peaks at 3445.24 cm^-1^ and 1636.78 cm^-1^ correspond respectively to ν(O–H) stretching vibrations and δ(H–O-H) bending vibration of the hydroxyl group of the adsorbed water presented in both samples, due to the physical adsorption of water from the atmosphere. Furthermore, the tiny peak in the range of 2368.15 cm^-1^ revealed the existence of carbon dioxide that exists in the air, as well as the peaks observed in the range of 1377 and 1079 cm^-1^ correspond to C-O stretching vibrations originating from surface-adsorbed carbonate species^28,33,38^.

#### PL Analysis

i. Bulk and milled Bi_2_O_3_ and CuO nanoparticles

**Fig. 7:**
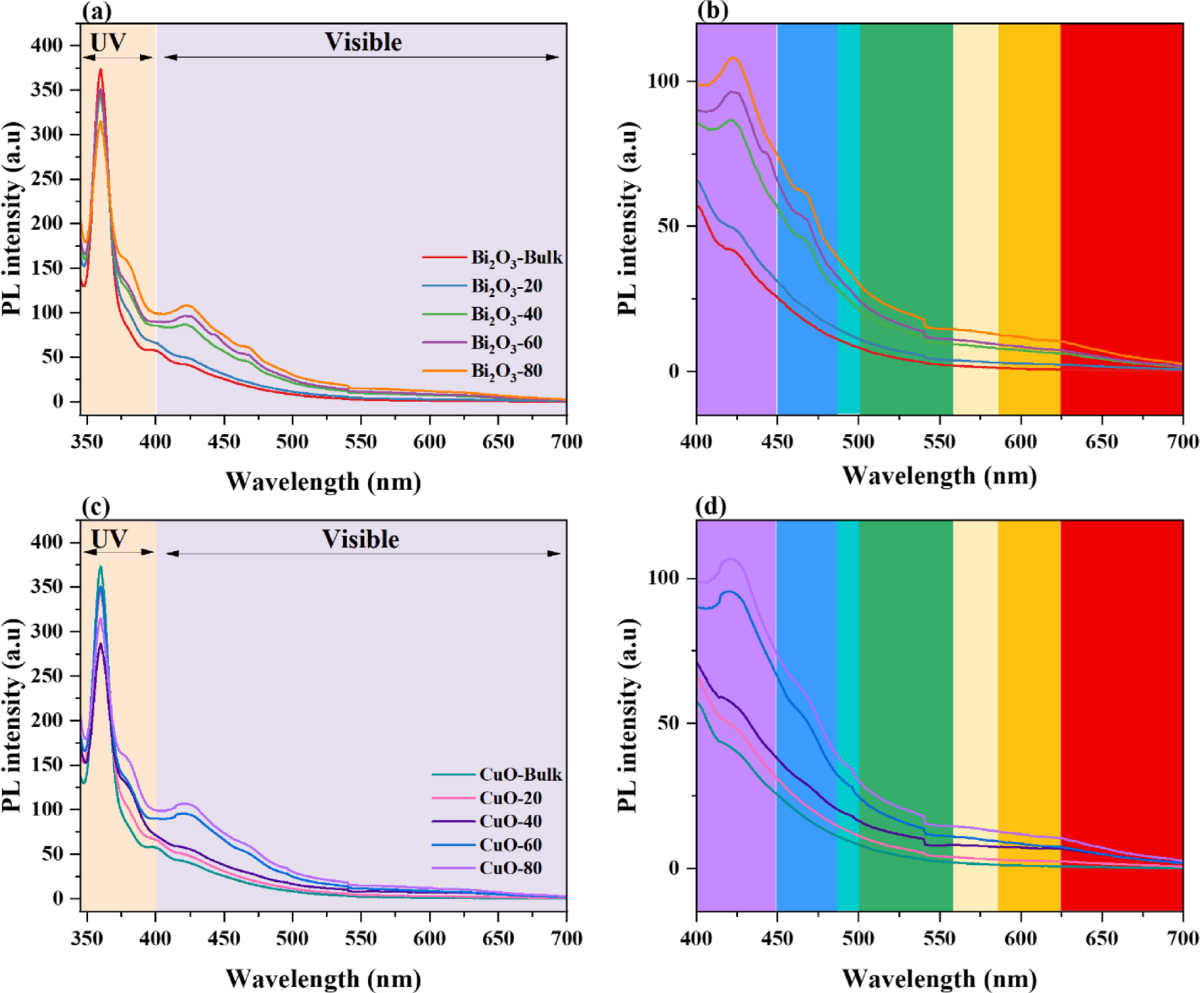
(**a** and **c**) PL spectra of the bulk and milled Bi_2_O_3_ and CuO nanoparticles and (**b** and **d**) zoom-in of the visible regions, respectively.

Using UV light with a wavelength of 330 nm, the prepared Bi_2_O_3_ nanoparticles’ emission dependency was first examined and tested in Fig. 7 (a). The emission range between 350 and 700 nm was taken into consideration to explain the structural faults. As the milling duration was extended to 80 min, a notable drop in the photoluminescence intensity was seen in the visible region (350–400 nm).

In general, PL quenching occurs as milling time increases due to the interaction of defect production, crystallinity reduction, and non-radiative recombination processes^39–41^. The PL spectra of Bi_2_O_3_ photoluminescence show several emission peaks that correspond to various defect states and electronic transitions. According to Zhang et al*.*^39^, excitonic recombination is the source of the near-band-edge (NBE) emission (~ 366–377 nm), which is especially noticeable in nanostructured Bi_2_O_3_. According to the literature^42^, defects connected to oxygen vacancies are responsible for peaks in the range of ~ 2.0 to 2.8 eV, while mid-gap states created by structural flaws are linked to violet (~ 440 nm) and blue (~ 470 nm) emissions as seen in Fig. 7(b). Bi^3+^-related charge transfer states are responsible for green emissions (~ 550 nm), whereas heavy lattice distortions are the source of deep-level defect emissions^41^. Defect-related emissions are enhanced in the milled samples due to higher oxygen vacancies and structural disorder, while the bulk Bi_2_O_3_ spectrum exhibits dominant band-edge emission. These findings reveal that the milling time variation improves the optical properties of Bi_2_O_3_ nanoparticles, making it a capable material for optoelectronic applications^43^.

Furthermore, the emission dependence of the produced CuO nanoparticles was initially investigated and demonstrated in Fig. 7 (c) using UV light with a wavelength of 330 nm. The emission range between 350 and 700 nm was considered to explain the structural defects. When the milling duration was extended to 80 min, the PL intensity significantly decreased. Defect formation, crystallinity reduction, and non-radiative recombination processes interact to cause PL quenching, which typically happens as milling time increases^39–41^. For every sample, the spectra show the existence of emission peaks. Strong UV emission bands, a sign of a near-band-edge (NBE) peak, were seen at ~ 358 nm and 375 nm. These UV peaks were attributed by Dagher et al.^44^ to the recombination of electrons and holes of the free excitons of CuO. At 406 nm, a little violet emission band was also identified, as shown in Fig. 7(d). Furthermore, as shown in the literature^45^, the violet range’s centered peak at 438 nm highlights the Cu vacancies (V_Cu_) transitions in CuO. The Cu interstitial (Cu_i_) either changed from Cu_i_ to the top of the valence band or from Cu_i_ to V_Cu_^46^. In the visible area, the blue emission section exhibited the highest deconvolution intensity, indicating a surplus of Cu interstitial defects relative to the other defects. In the bulk CuO spectrum, band-edge emission is prominent, but increased oxygen vacancies and structural disorder in the milled samples enhance defect-related emissions. According to these results, CuO nanoparticles’ optical characteristics are enhanced by the milling time variation, which makes it a suitable material for near-UV white light-emitting devices^47^.

ii. Bi_2_O_3_-CuO nanocomposite

**Fig. 8:**
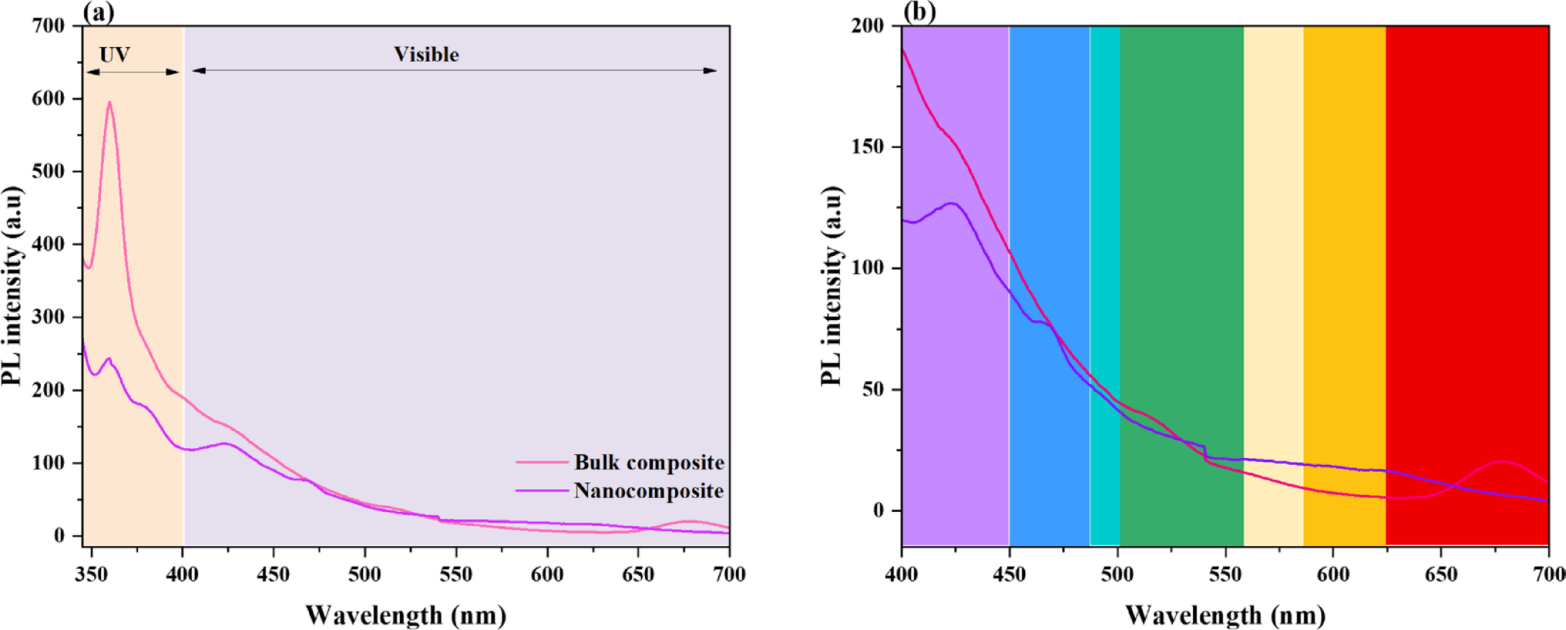
(**a**) PL spectra of (70 wt% Bi_2_O_3_/30 wt% CuO) bulk and nano composites and (**b**) zoom-in of the visible region.

The PL spectra of bulk composite and nanocomposite samples are shown in Fig. 8 (a). Deconvoluted peaks reveal different emission contributions, with a zoomed-in view of the visible region displayed in Fig. 8(b). A prominent emission peak at lower wavelengths in the nanocomposite spectrum indicates a dominant radiative recombination mechanism, which is most likely caused by inherent defects or near-band-edge emissions. A wider emission profile with several deconvoluted peaks is shown in the bulk composite spectrum, which suggests a greater number of defect-related recombination routes as well as potential contributions from oxygen vacancies, structural distortions, or impurity states. The observed difference between the two spectra indicates that variations in the PL intensity and spectral distribution are caused by material alterations, such as milling time, doping, or compositional changes, which affect the defect density and carrier dynamics. Peak positions may broaden and shift, which could potentially indicate improved non-radiative recombination or quantum confinement effects. These are important aspects in adjusting the material’s optical characteristics for certain uses. A number of variables account for the sharp variations in PL, including (i) defect density: high-energy ball milling increases microstrain and decreases particle size, which results in a higher density of structural flaws and oxygen vacancies. As recombination centers, these change emission characteristics and quench PL intensity^48^. (ii) Quantum confinement: The confinement of charge carriers alters the electronic band structure as particle sizes go closer to the nanoscale. This can change recombination dynamics and shift emission energies, which helps to explain some of the variations between bulk and nanoscale oxides^49^. (iii) Polypropylene interfacial effects: Nanoparticles in nanocomposites interact with the PP matrix more strongly and have a higher surface-to-volume ratio. This promotes interfacial charge transfer and energy dissipation pathways, which further influence PL behavior compared to bulk fillers^50^.

#### Scanning electron microscopy and energy dispersive X-ray spectroscopy (SEM–EDX)

i. Bulk and milled Bi_2_O_3_ and CuO nanoparticles

**Fig. 9:**
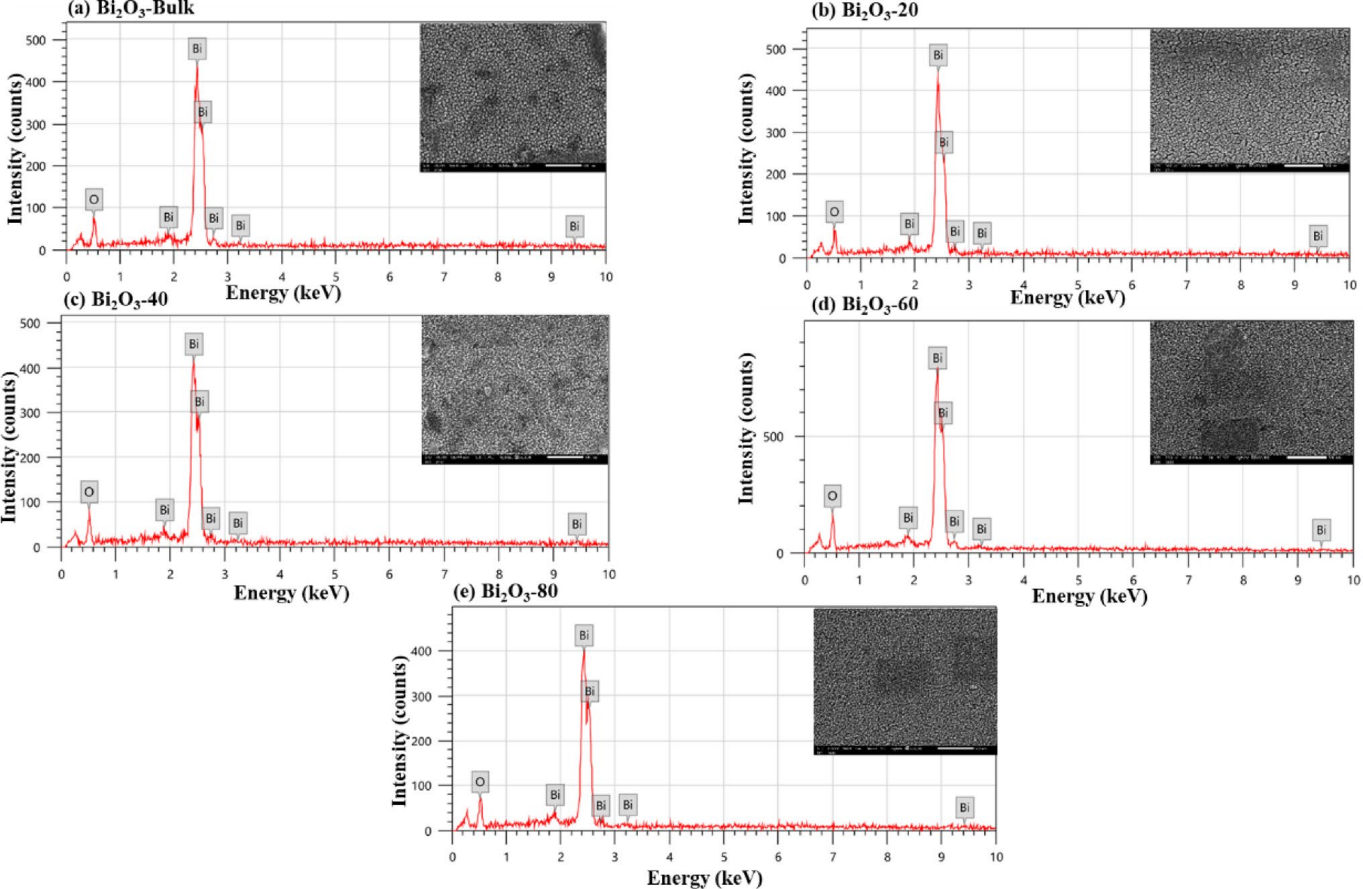
(**a-e**) EDX analysis of the bulk and milled Bi_2_O_3_ samples with the insets of SEM images.

**Fig. 10:**
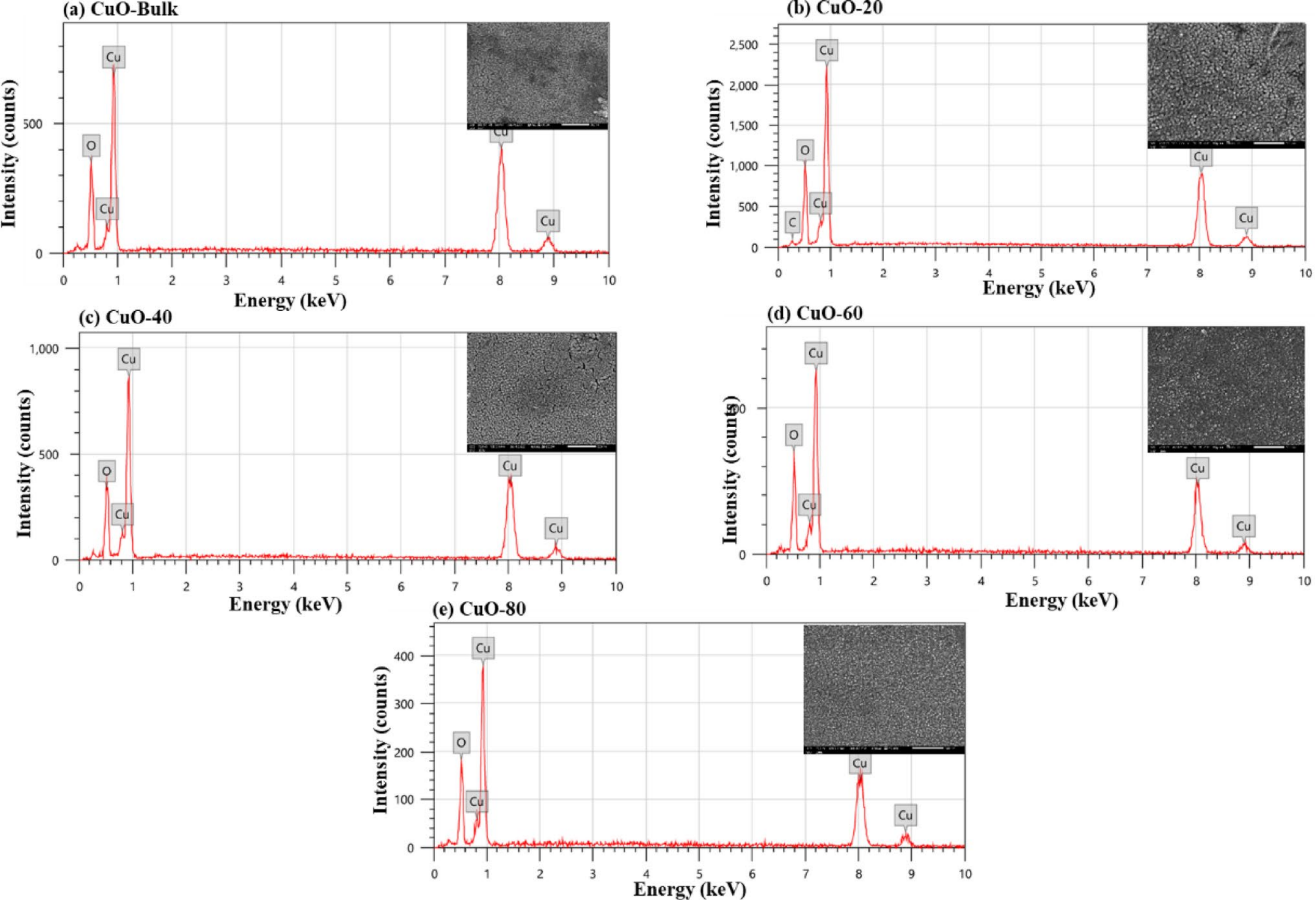
(**a-e**) EDX analysis of the bulk and milled CuO samples with the insets of SEM images.

**Fig. 11:**
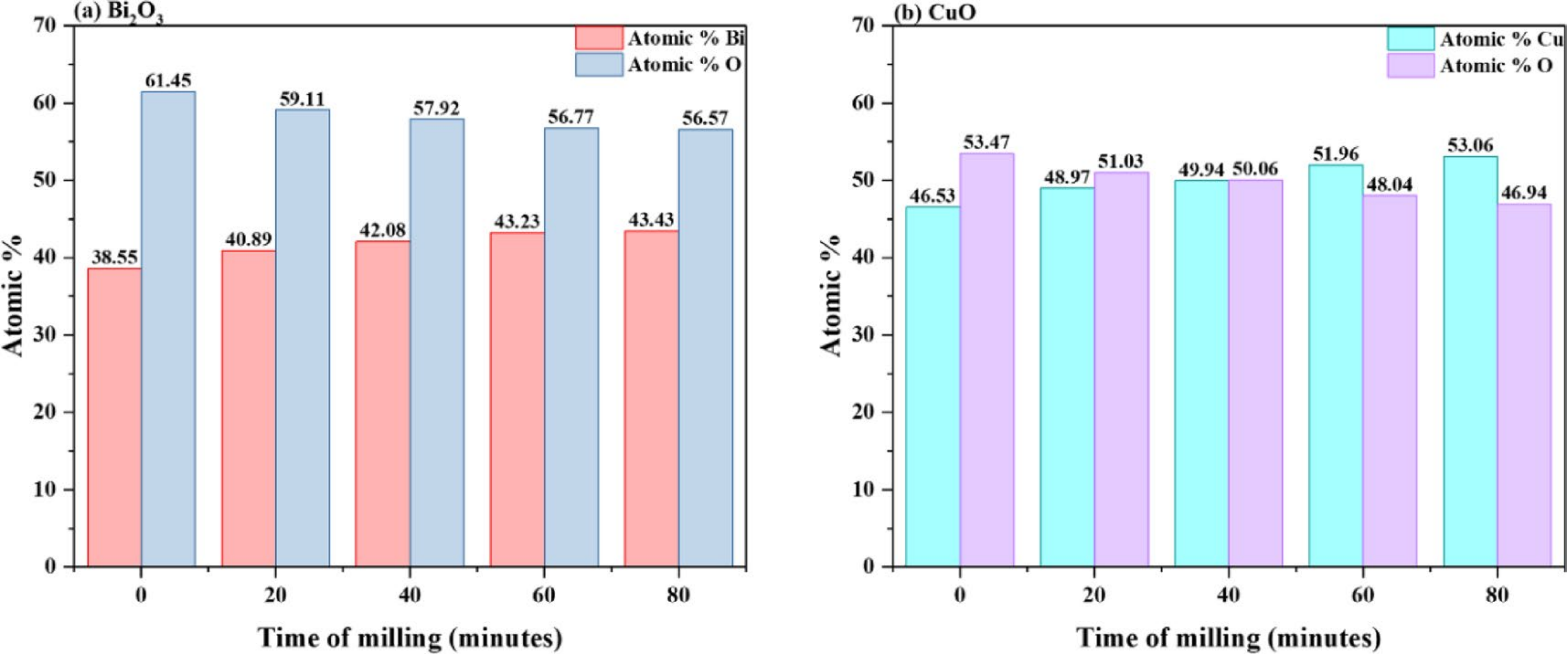
EDX results of the atomic% distribution of (**a**) Bi and O elements in bulk and milled Bi_2_O_3_ samples, (**b**) Cu and O elements in bulk and milled CuO samples.

The SEM pictures and EDX analysis of bulk and milled Bi_2_O_3_ at various milling times (20, 40, 60, and 80 min) are displayed in Fig. 9(a-e). While milled Bi_2_O_3_ exhibits the existence of distinctly fine aggregates and decreasing particle sizes as the milling duration reduces in comparison to bulk Bi_2_O_3_, the SEM picture of bulk Bi_2_O_3_ showed that Bi_2_O_3_ particles are in the form of minute grains of diverse shapes and sizes. Additionally, peaks in the EDX elemental analysis verified that the Bi and O elements were the primary constituents of the various Bi_2_O_3_ samples throughout the spectroscopic procedure. The EDX results of the atomic% distribution of Bi and O in each of the studied Bi_2_O_3_ samples are shown in Fig. 11(a). The quantitative analysis confirms the presence of Bi_2_O_3_ and shows the close atomic ratios of Bi and O, which is 2:3. Similarly, the SEM images of CuO are shown in Figs. 10 (a-e) appear like irregular sphere particles with confused shapes and sizes according to the different milling times of CuO. The chemical composition of the CuO samples is shown in Figs. 10 (a-e) by an EDX analysis confirmed the existence of its main elements Cu and O. Furthermore, the quantitative atomic% distribution of Cu and O in CuO samples at different milling times are presented in Fig. 11 (b), Cu and O were only appeared in all investigated samples and show the close atomic ratios of Cu and O which is 1:1.

The very small deviation of the atomic ratios of both Bi_2_O_3_ and CuO may be attributed to a lack or excess of the surface adsorption of oxygen or the overestimation of the lighter elements detected by EDX analysis. The apparent increase in metal atomic % and decrease in oxygen % after milling is due to surface oxygen loss and reduced sensitivity of EDX to lighter elements. High-energy collisions during milling create oxygen vacancies and local nonstoichiometry, while the EDX detector tends to underestimate light elements such as oxygen^51^.

ii. Bi_2_O_3_-CuO nanocomposite

**Fig. 12:**
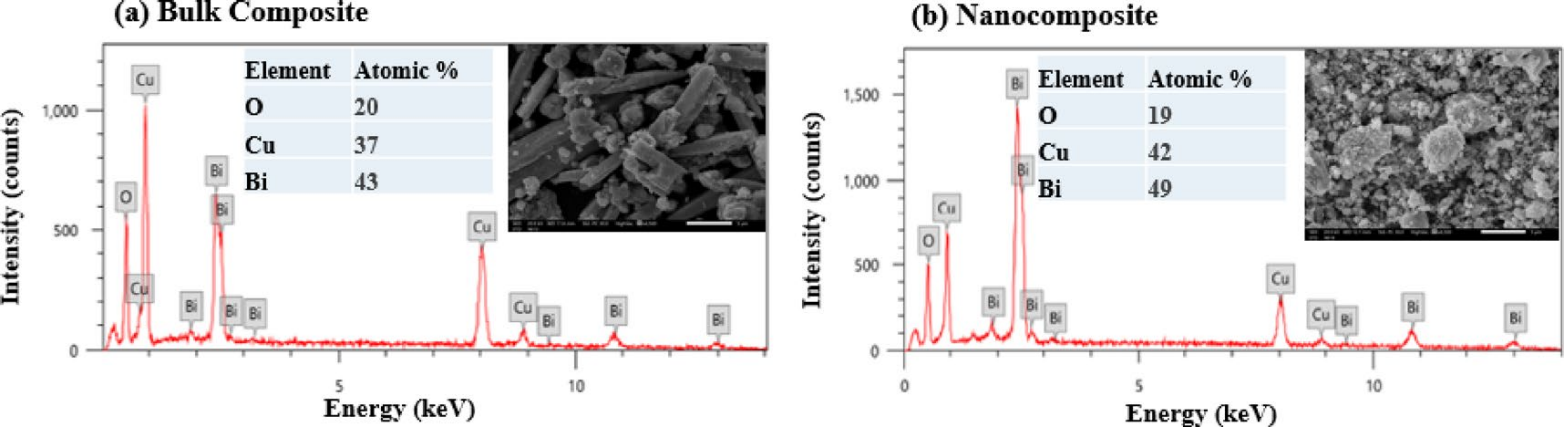
EDX analysis with the insets of SEM images of (70 wt% Bi_2_O_3_/30 wt% CuO) (**a**) bulk and (**b**) nanocomposite.

Figure 12 shows the SEM images and EDX analysis of 70% Bi_2_O_3_ and 30% CuO blend fillers used for both bulk and nano (Bi_2_O_3_/CuO) composites. Bi_2_O_3_ particles appear cubic-like, while those of CuO show spherical shapes. In contrast to bulk (Bi_2_O_3_/CuO) blended fillers, the SEM images demonstrate the presence of distinctly fine aggregates and lower particle sizes for nano (Bi_2_O_3_/CuO) fillers.

Furthermore, for both bulk and nano-blended fillers, the EDX elemental analysis revealed peaks verifying the presence of Bi, Cu, and O elements. The Bi peaks appeared to be increased in the nanofiller in contrast to Cu, indicating that Bi nanoparticles occupy the largest surface area of the quantitative analysis due to their higher weight fraction compared to Cu.

#### Transmission electron microscopy (TEM)

**Fig. 13:**
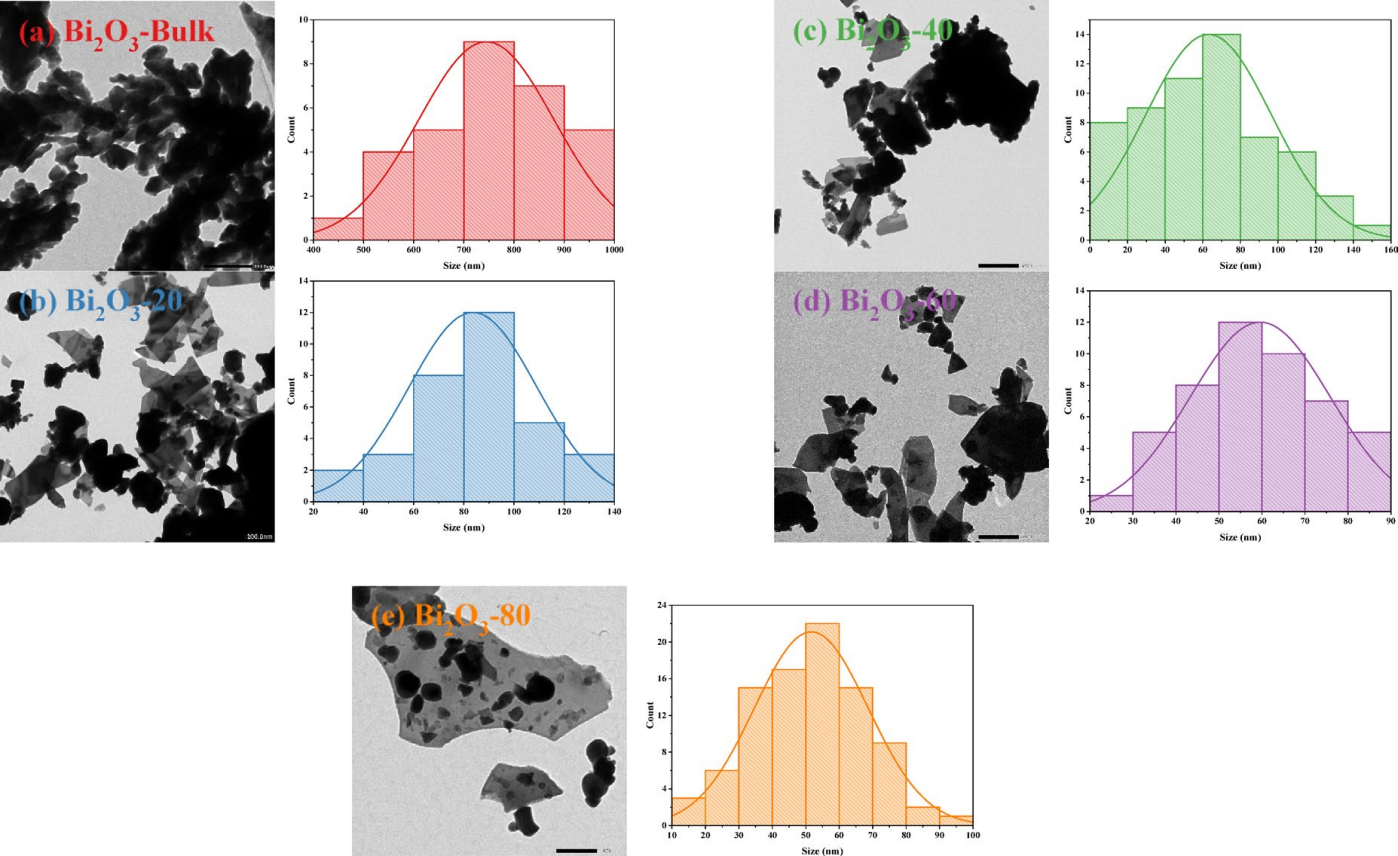
(**a-e**) TEM micrographs and particle size histograms of bulk and milled Bi_2_O_3_ samples.

**Fig. 14:**
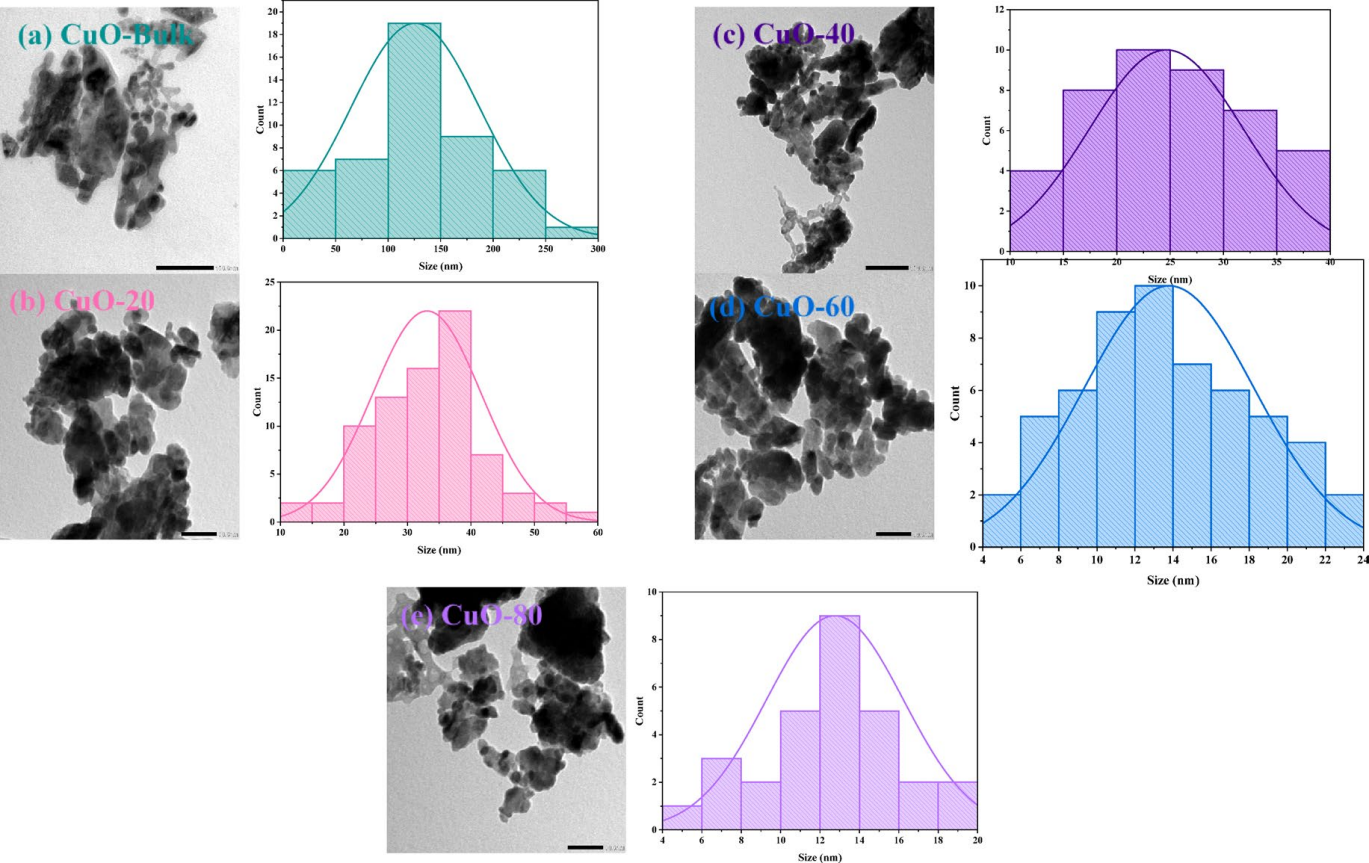
(**a-e**) TEM micrographs and particle size histograms of bulk and milled CuO samples.

Complementary TEM micrographs and the particle size histograms of bulk and milled Bi_2_O_3_ and CuO at different milling times (20, 40, 60, and 80 min) are described in Figs. 13 (a) to (e) and Figs. 14 (a) to (e), respectively. The morphology of Bi_2_O_3_ showed a cubic-like like, while that of CuO showed spherical shapes. As well as the presence of agglomeration and non-uniform particles was detected in both samples. The high surface free energy leads to heavily agglomerated particles, which may result in magnetic contact between the particles^52,53^. Using ImageJ software, a Gaussian distribution was used to measure the particle size distribution based on the length scale. The average particle size obtained for Bi_2_O_3_-Bulk, Bi_2_O_3_-20, Bi_2_O_3_-40, Bi_2_O_3_-60, and Bi_2_O_3_-80 were found to be approximately 743 nm, 83 nm, 61 nm, 58 nm, and 52 nm, while for CuO-Bulk, CuO-20, CuO-40, CuO-60, and CuO-80 were found to be approximately 126 nm, 33 nm, 25 nm, 14 nm, and 13 nm, respectively.

**Fig. 15:**
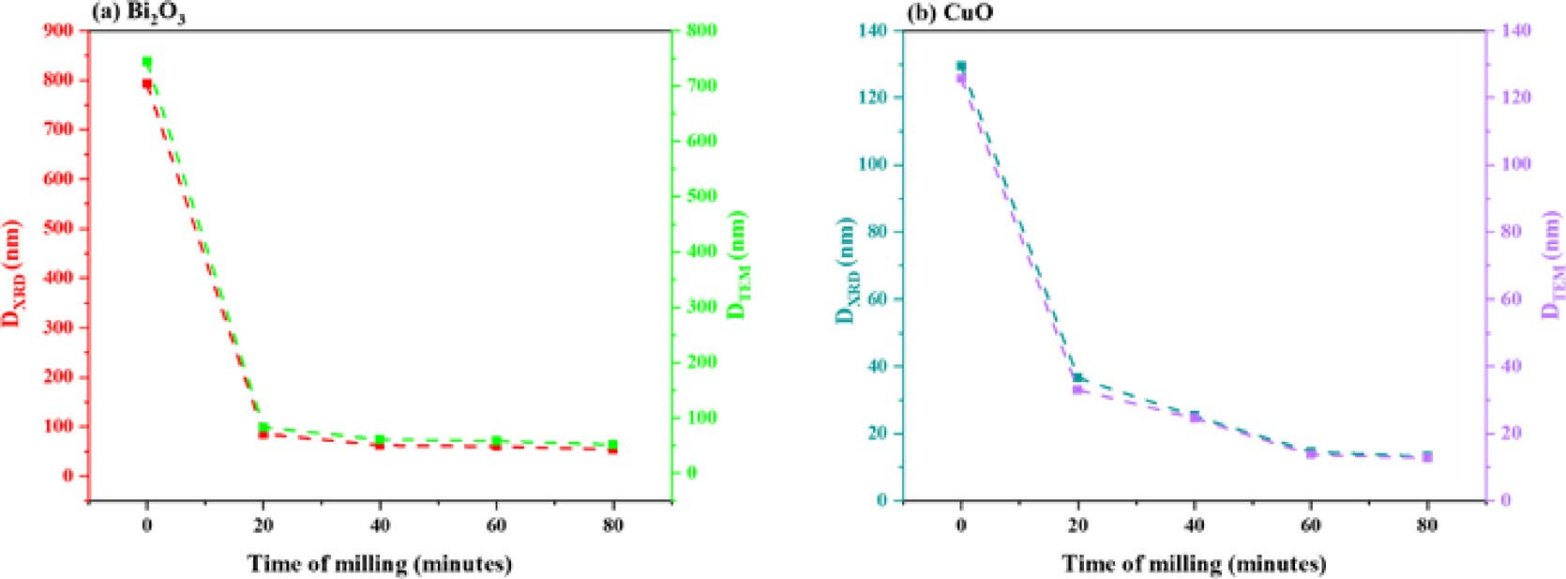
The average size extracted from TEM and XRD for bulk and milled (**a**) Bi_2_O_3_ and (**b**) CuO samples.

The findings roughly match the average size that was taken from the milled Bi_2_O_3_ and CuO XRD patterns, as shown in Figs. 15(a) and (b). It shows how the crystallite size (D_XRD_) and particle size (D_TEM_) of Bi_2_O_3_ and CuO drop as the ball milling duration increases. There is a steep decline in the first 20 min, followed by a saturation trend. The high mechanical energy used in milling, which fragments particles and refines crystallites, is responsible for this quick initial drop. However, after prolonged milling, both sizes go closer to a steady state because of a balance between cold welding and particle fracture, where agglomeration prevents further size reduction. Furthermore, as the system gets closer to a thermodynamic limit of grain refinement, further size reduction becomes energetically unfavorable, and extended milling generates structural strain and flaws. This convergence in TEM and XRD size results was observed by Khairnar et al.^54^ who synthesized Bi_2_O_3_ by the sol–gel method, and Vijayalakshmi et al.^55^ who prepared Bi_2_O_3_ by the co-precipitation method, as well as El Sayed et al.^21^ that prepared CuO by the co-precipitation method.

### Structural and thermal properties of PP and PP incorporated with bulk and nanofillers

#### Structural properties using XRD

**Fig. 16:**
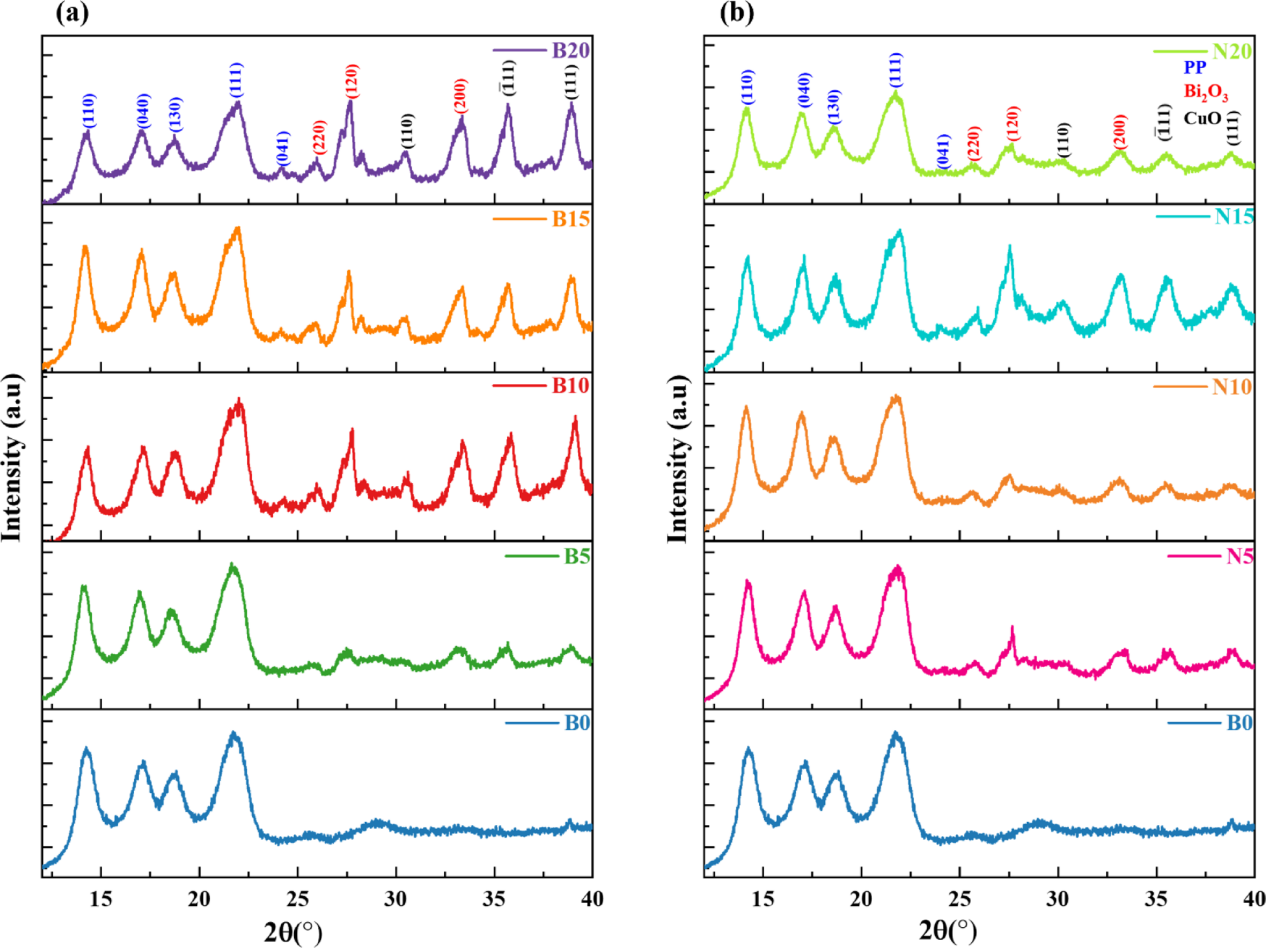
XRD patterns for (**a**) PP/bulk composites and (**b**) PP/nanocomposites.

Figures 16 (a and b) show the XRD patterns of PP and PP combined with filler weight fractions of 5%, 10%, 15%, and 20% (Bi_2_O_3_/CuO) Bulk (B), and (Bi_2_O_3_/CuO) nanocomposite (N). The XRD patterns of PP showed the presence of both crystalline and amorphous regions. They exhibit two strong reflection peaks at 14.19^˚^ and 21.75^˚^, surrounded by two less intensive peaks at 17.02˚ and 18.53^˚^ related to (110), (040), (130), and (111) lattice planes, respectively. These peaks of the crystalline region are compatible with studies that indicate the monoclinic α-phase of PP^56,57^. The XRD patterns of the synthesized (Bi_2_O_3_/CuO) composites in Figs. 16(a and b) show the blends of the characteristic peaks of PP and the different bulk and nanoparticle sizes of (Bi_2_O_3_/CuO).

The XRD patterns of (Bi_2_O_3_/CuO) Bulk (B) composites showed no alteration in the position peaks, confirming that there is no effect on either the chemical or crystal structure of PP^58^. Whereas, the intensity was changed depending on the weight fraction of (Bi_2_O_3_/CuO) bulk(B) in the PP composite. This behavior is illustrated in Fig. 16(a), where the corresponding intensity of the PP characteristic lattice planes (110), (040), (130), and (111) declined when the wt. % increases. While the three characteristic lattice planes (220), (120), and (200) of bulk CuO and the three characteristic lattice planes (110), (111) and (111) of bulk Bi_2_O_3_ appeared more clearly as the filler weight fraction increases indicating fine dispersion of the filler inside the polymer. Analogous observations are shown in Fig. 16(b) for (Bi_2_O_3_/CuO) nanocomposite (N) composites, where the characteristic lattice planes (110), (040), (130) and (111) of PP declined with increasing the filler weight fraction, while the three characteristic peaks of nano CuO and nano Bi_2_O_3_ appeared more clearly as the weight fraction increases. Furthermore, there was a noticeable variation in the XRD patterns of (Bi_2_O_3_/CuO) composites at the same weight fraction. When the filler particle size dropped from bulk (B) to nanoparticle (N), this difference manifested as a broadening and weakening of all peaks. These modifications show a drop in the degree of crystallinity of the composites, likely due to the presence of microstrain and the decrease in Bi_2_O_3_/CuO crystallite size^59^.

The main X-ray diffraction pattern occurs differently for each separate phase of (Bi_2_O_3_/CuO) composites, as illustrated in Figs. 16(a and b). This demonstrates that the metal oxides are physically adsorbed on the surface polymer matrix, as was also noted in a study^60^ on HDPE that contained bulk lead oxide and various weight fractions (10% and 50%) of nanoparticles. They discovered that the chemical and crystal structures of HDPE are unaffected by the addition of PbO.

#### Thermal properties using thermal gravimetric analysis (TGA)

**Fig. 17:**
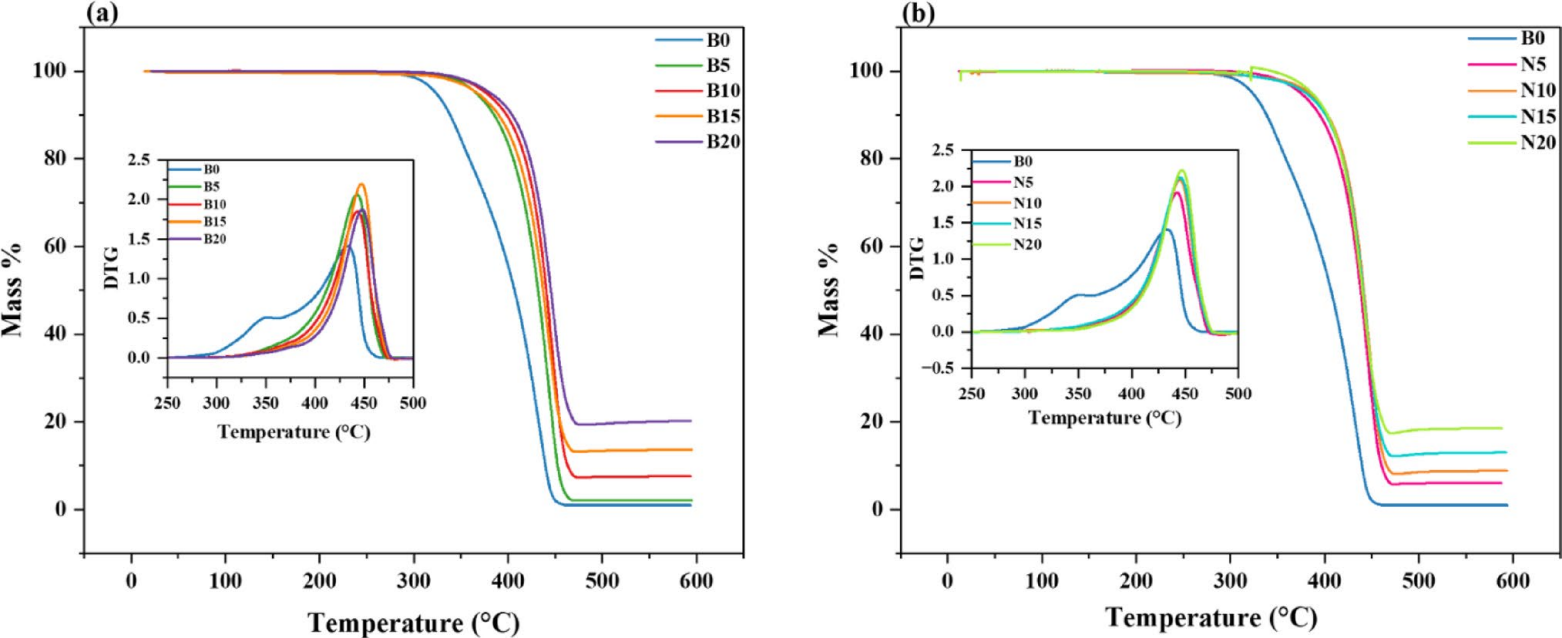
TGA curves for (**a**) PP/bulk composites and (**b**) PP/nanocomposites. The insets represent the DTG curves for the same group of samples.

Thermogravimetric analysis (TGA) and derivative thermogravimetric analysis (DTG) were used to assess the thermal stability of PP. The impact of bulk composites (B5–B20) and nanocomposites (N5–N20) on the thermal properties of PP was compared and shown in Figs. 17(a and b). The relevant derivatives (DTG) are displayed in the insets of Figs. 17. T_5%_ and T_50%_ temperatures, which correlate to a 5% and 50% weight loss of the studied samples, along with the percentage of residual mass, were computed and presented in Table 2. There was just one degradation phase visible in the TGA curves for all samples. PP decomposition (B0) started at 324 °C and continued until it completely broke down at 450 °C, leaving a 0.97% residual mass. Tipboonsri et al.^61^ found similar results, suggesting that the TGA data showed that the thermal degradation of PP started at about 320 °C.

**Table 2:** Thermal degradation properties of PP polymer composites with bulk and nanocomposites.

Sample	TGA results			DTG results	
	T_5%_ (°C)	T_50%_ (°C)	Residual mass %	Decomp. T (°C)	Decomp. Rate (%/°C)
B0	324	406.77	0.97	433	1.41
B5	366	432	2.16	442	2.06
B10	377	441	7.57	443	1.84
B15	368	437	13.63	447	2.19
B20	381	445	20.23	448	1.87
N5	375	438	6.02	442	1.91
N10	384	441	8.82	445	2.09
N15	380	443	13.02	446	2.12
N20	387	446	18.57	447	2.22

When compared to the pure polymer (B0), the thermal stability of PP is greatly increased by the addition of bulk reinforcements (B5–B20). Due to enhanced thermal resistance, the initial degradation temperature (T_5%_) rises from 324 °C (B0) to 381 °C (B20), suggesting a delayed degradation onset. This increase can be attributed to the bulk filler’s ability to act as a heat barrier, which reduces polymer chain mobility and delays breakdown. The following mechanisms are more likely to be responsible for the composites’ increased thermal resistance: (i) Barrier effect: When oxide particles are evenly distributed, they might form a convoluted channel that inhibits the flow of oxygen and volatile degradation products into the polymer matrix, postponing decomposition^62^. (ii) Formation of char layer: During thermal deterioration, metal oxides can encourage the development of a compact protective char layer that serves as a physical barrier against heat and mass transport^63^. (iii). Interfacial interactions: Interfacial interactions between PP and oxide surfaces might limit chain mobility locally, improving stability, even if they are weaker than in carbonaceous fillers^64^. The addition of carbon black (CCB) particles caused ultra-high molecular weight polyethylene (UHMWPE) to break at higher temperatures, according to Cheng et al.^65^. This enhancement was attributed to the interfacial interaction between UHMWPE granules and polar CCB particles, which substantially improved thermal stability and inhibited heat transfer inside the polymer chains^65^. In the case of nanocomposites (N5–N20), a similar trend is observed. The initial decomposition temperature (T_5%_) increases more prominently from 324 °C (B0) to 387 °C (N20), slightly surpassing the thermal stability achieved with bulk fillers. This suggests that the nano-sized fillers provide even greater stabilization, likely due to their higher surface area and stronger interaction with the polymer matrix. As claimed by Majka et al.^66^, well-dispersed nanoparticles offer superior heat shielding and enhance polymer-filler interfacial bonding, which leads to enhanced thermal resistance.

The trend continues with the T_50%_ values. For bulk composites, T_50%_ increases from 407 °C (B0) to 445 °C (B20), further confirming the improved thermal durability. The ability of the bulk fillers to form a protective char layer and reduce the transfer of thermal energy plays a major role in this enhancement. Dasari et al.^67^ also reported similar behavior in flame-retardant polymer composites, where bulk reinforcements enhanced thermal resistance through improved char formation. Nanocomposites (N5–N20) also show an obvious rise in T_50%_, increasing from 407 °C (B0) to 446 °C (N20). This behavior is likely due to the creation of a finely dispersed network that restricts polymer chain mobility and impedes the diffusion of volatile decomposition products. Singh et al.^68^ stated that nanofillers such as nano-clay and carbon nanotubes hinder radical transport, promoting slower degradation.

Additionally, both types of composites show an increase in residual mass at the end of degradation. For bulk composites, it rises from 0.97% (B0) to 20.23% (B20), while for nanocomposites, it reaches 18.57% (N20). Thus, it indicates that the composite structure encourages char production and inhibits full breakdown. Decomposition temperatures change to higher values, as indicated by the DTG data, with decomposition temperature rising from 433 °C (B0) to 448 °C (B20) for bulk composites and to 447 °C (N20) for nanocomposites. This supports the overall trend of increased thermal resistance with filler incorporation. But rather than consistently slowing down heat degradation, the decomposition rate varies, suggesting that the filler may have an impact on the degradation mechanism. Thus, bulk composites offer greater degradation temperatures and better char formation, which is consistent with research on flame retardancy in polymer composites by Dasari e*t al.*^67^. This also suggests that the contributions of bulk and nanoscale fillers to thermal resistance are comparable. The slight increase in residual carbon alone cannot account for the increase in degradation onset from 433 °C to around 447–448 °C with filler addition. Rather, we credit the enhanced thermal resistance to a confluence of factors, including the development of a more cohesive oxide-reinforced char that acts as a barrier against heat and mass transfer, modifications to the degradation pathways at oxide surfaces (allowing for radical scavenging or dehydration/crosslinking), and a change in the rate-controlling mechanism from chemical bond scission to diffusion through the protective layer.

The decomposition rate of bulk composites increases from 1.41 to 1.87%/°C for B20 sample. In contrast to bulk composites, the decomposition rate in nanocomposites grows more steadily (from 1.41 to 2.22%/°C), indicating a more regulated and consistent degradation process. These results highlight how nanofillers, which function as thermal barriers and restrict polymer chain mobility, improve the thermal stability of polymer composites.

To fully understand the degrading behavior of composites in comparison to pure PP, kinetic analysis of the thermal degradation process for pure PP, PP/bulk composites, and PP/nanocomposites has been carried out. Using Eq. (1) and the approximations provided by the following equation, the activation energy, $E$, can be computed^69^.

2 $$ln(\mathit{ln}\left(\frac{m}{{m}_{0}}\right))=-\frac{E}{R}\left(\frac{1}{T}\right)+A,$$

where $R$ is the ideal gas constant or 8.314 J/mol⋅K, $A$ is the frequency of molecular collisions leading to decomposition, and $m$ and ${m}_{0}$ are the masses of the sample at a specific temperature and the original sample mass, or 100%, respectively.

**Fig. 18:**
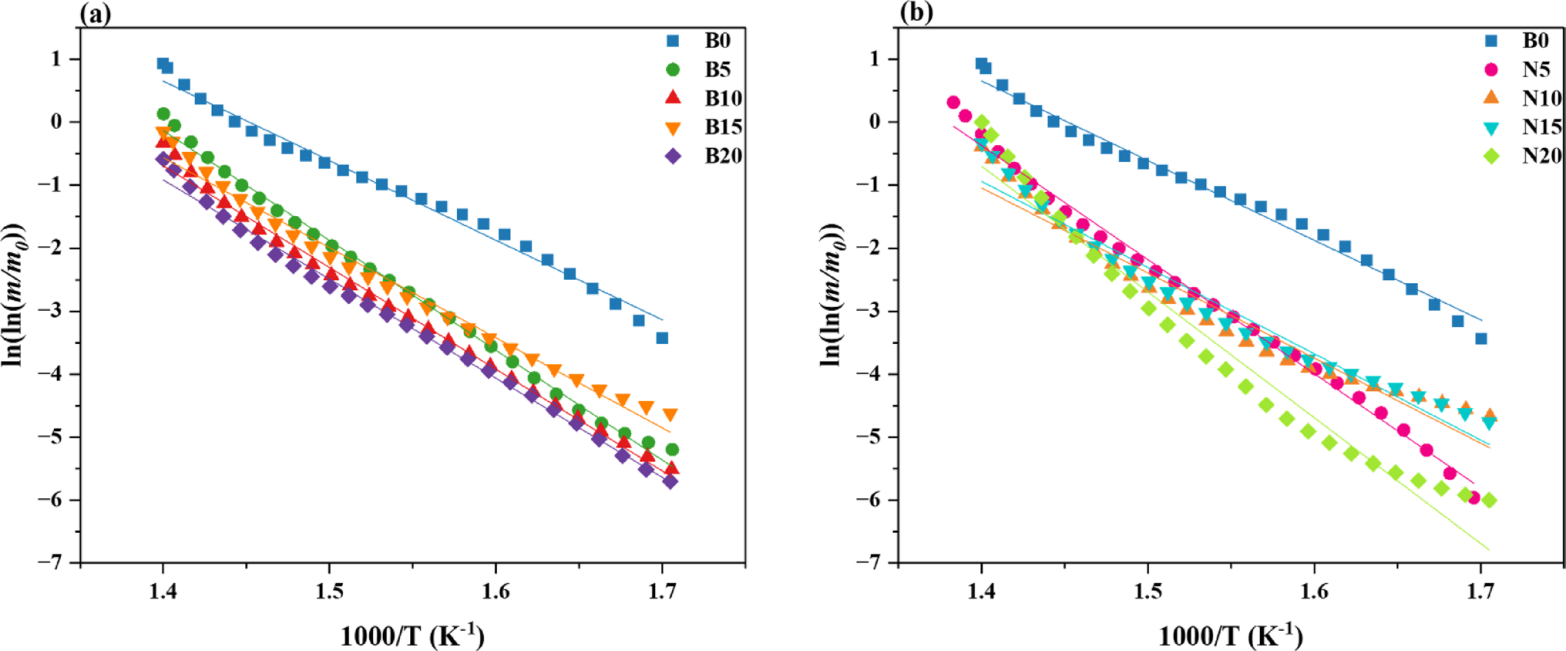
$ln(\mathit{ln}\left(\frac{m}{{m}_{0}}\right))$ against $\frac{1}{T}$ of (**a**) PP/bulk composites and (**b**) PP/nanocomposites.

**Fig. 19:**
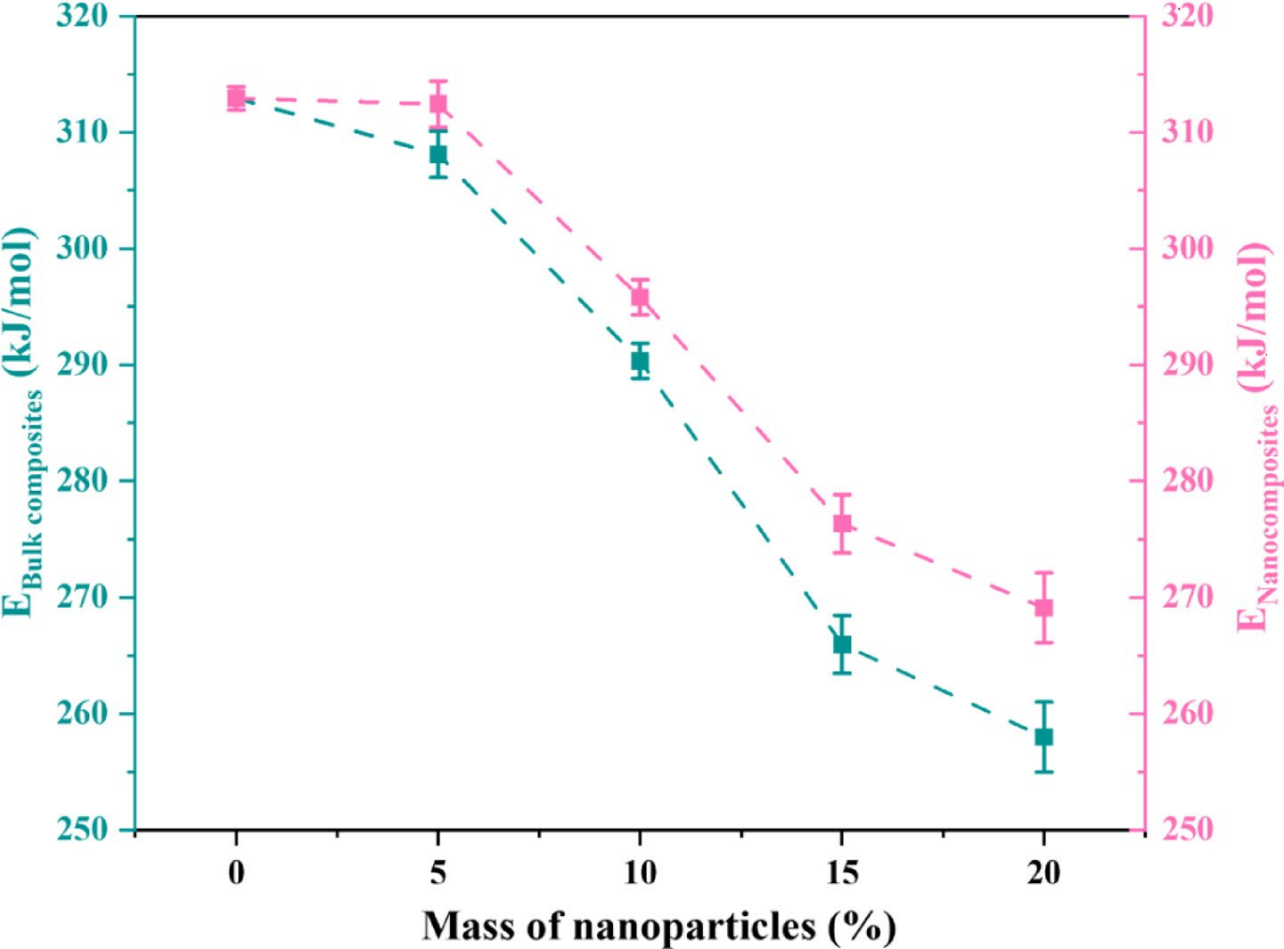
Variation of activation energy $(E)$ for PP polymer composites with bulk and nanocomposites.

TGA data of the examined PP/bulk and PP/nanocomposites were used for this purpose. Consequently, the $ln(\mathit{ln}\left(\frac{m}{{m}_{0}}\right))$ against $\frac{1}{T}$ plots shown in Figs. 18 (a and b) provide a straight line with a slope of $-\frac{E}{R}$. Consequently, the activation energy was computed using the slope. Table 3 presents all the kinetic parameters of the prepared samples. It is found that the lines measured at different weight fractions of bulk composites and nanocomposites are approximately parallel to each other. Furthermore, the linearity correlation coefficient (R^2^) for determining each activation energy is higher than 0.99, indicating that the degradation process is well described by the used kinetic model. Also, the calculated activation energy of each sample is presented in Fig. 19.

**Table 3:** Kinetic parameters calculated for PP polymer composites with bulk and nanocomposites.

Sample	-E/R	A	R^2^
B0	37.64	56.57	0.999
B5	37.06	53.38	0.994
B10	34.92	49.81	0.992
B15	31.99	45.41	0.992
B20	31.03	44.15	0.996
N5	37.58	54.49	0.989
N10	35.58	51.01	0.991
N15	33.24	47.72	0.982
N20	32.37	47.98	0.999

Table 3 illustrates a distinct declining trend in $-\frac{E}{R}$ and $A$ for bulk composites (B5–B20) as filler content rises. According to the activation energy studies, this implies that bulk fillers reduce the energy barrier for deterioration. According to Fig. 19, filler-induced heterogeneities that serve as heat transfer sites and speed up degradation may be the cause of the lower activation energy. Weak connections in polymer composites, such as head-to-head, hydroperoxy, and peroxy structures, which easily decompose at very low temperatures to produce radicals that aid in the degradation process at higher temperatures, are associated with polymer degradation^70^. The residual mass readings from TGA, however, show better char formation despite the drop in $-\frac{E}{R}$, which helps to increase thermal protection.

Similar trends are observed for nanocomposites (N5–N20), albeit for equivalent filler contents, the $-\frac{E}{R}$ values are comparatively larger than those of bulk composites. This gives credence to the idea that nanofillers improve thermal barriers by limiting heat transfer and polymer chain mobility, which postpones breakdown. Particularly at greater nanofiller content, the $A$ factor exhibits a declining trend, suggesting a decreased probability of spontaneous degradation processes (Table 3).

Figure 19 shows that bulk-filled PP exhibits a more noticeable drop in $E$ when compared to nanocomposites, suggesting that bigger filler particles facilitate thermal breakdown more successfully than milled nanoparticles. This may be because bulk composites have fewer microstructural discontinuities and interfacial interactions, which promote heat transport and polymer degradation. The idea that nano-sized fillers improve the thermal barrier effect by creating a more uniform dispersion and limiting heat-induced polymer degradation is supported by the fact that nanocomposites maintain a comparatively higher $E$. According to similar patterns shown in the literature, nano-fillers such as carbon-based materials and clay strengthen the interfacial interaction between the polymer and the filler, increasing the thermal stability of the polymer^67^. Overall, nanocomposites improve thermal stability by slowing down the degradation process through higher interfacial adhesion and heat dissipation effects, whereas bulk fillers lower the activation energy more quickly.

#### Thermal properties using differential scanning calorimetry (DSC)

**Fig. 20:**
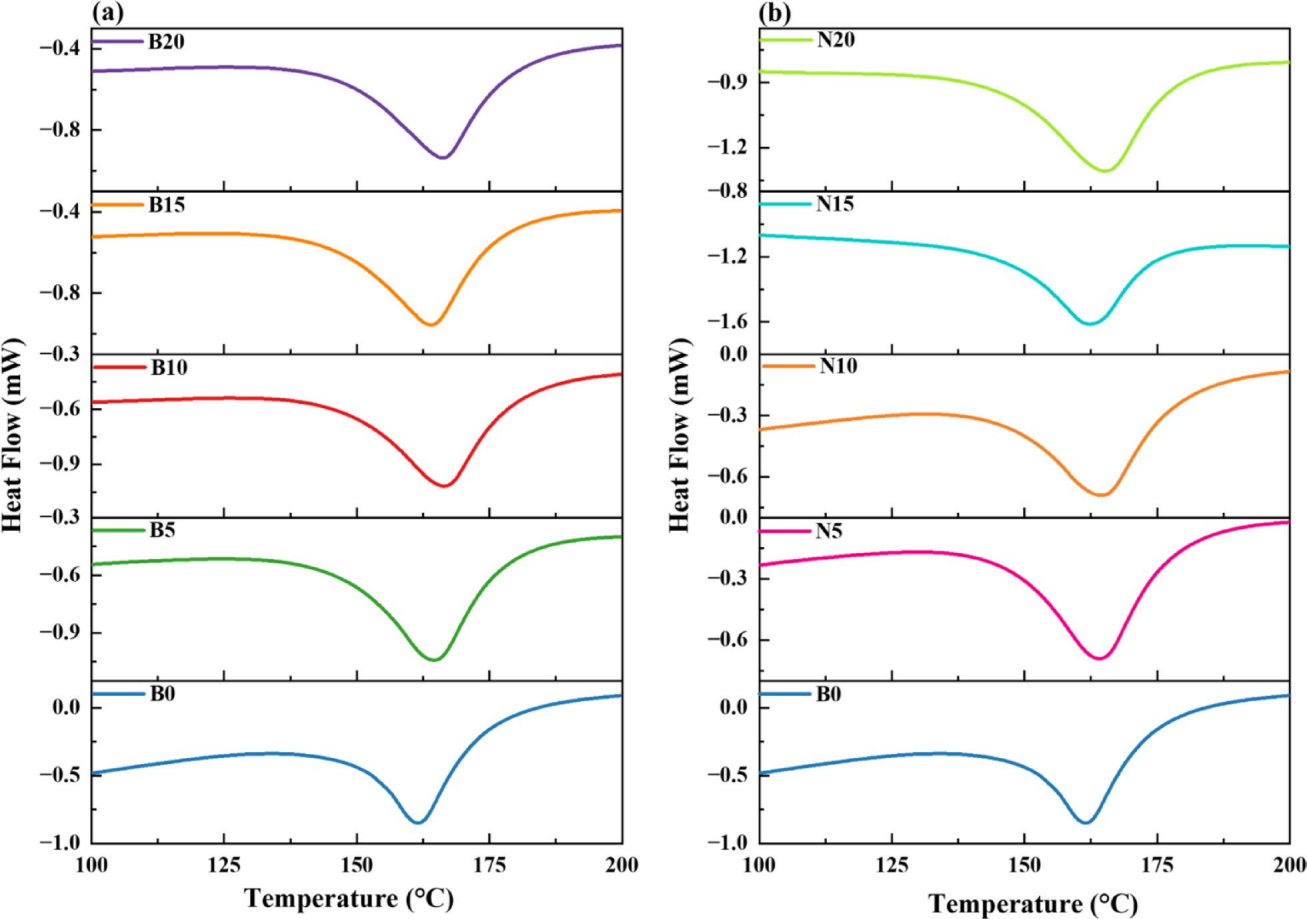
DSC heating curves for (**a**) PP/bulk composites and (**b**) PP/nanocomposites.

A thorough understanding of the effects of bulk (B5–B20) and milled nanoparticle-based (N5–N20) fillers on the thermal behavior of PP is offered by the Differential scanning calorimetry (DSC) thermograms and associated thermal transition data. Figure 20 (a and b) shows the DSC curves of PP/bulk composites and PP/nanocomposites, respectively.

A popular semicrystalline polymer is polypropylene (PP). Both amorphous and semicrystalline polymers undergo a glass transition (${T}_{g}$) when the temperature rises. Nevertheless, semicrystalline polymers like PP undergo crystallization and melting, whereas amorphous polymers only demonstrate the glass transition. Even though PP has a glass transition temperature, it is not very high and is frequently not discernible in thermal investigations. The ${T}_{g}$ of PP is between −20 °C and 0 °C, and beyond this range, PP becomes brittle because of decreased molecular mobility, according to Kang et al*.*^71^. Likewise, Collar et al*.*^72^, state that the degree of polymerization raises the ${T}_{g}$ of atactic polypropylene. With no detectable ${T}_{g}$ within the studied temperature range of 100–200 °C, the thermal curves in this work mainly show transitions associated with melting and crystallization. The behavior of isotactic polypropylene, which has a ${T}_{g}$ of about −10 °C, which is significantly lower than the scanning range used, is in line with this. Table 3 lists the DSC measurement findings, which include the melting temperature at onset, peak, and endset.

The melting temperature of the control sample (B0) is 162 °C, whereas its ${T}_{onset}$ is 151 °C and its ${T}_{Endset}$ is 171 °C. A general trend of rising ${T}_{m}$, ${T}_{onset}$, and ${T}_{Endset}$ can be seen with the inclusion of bulk and nanofillers, especially in the nanocomposites series. From 151 °C for PP (B0) to 144 °C for B20, the ${T}_{onset}$ is somewhat lowered with the addition of bulk fillers (B5 to B20). This decrease suggests that the commencement of crystalline melting happens earlier when the filler is incorporated, most likely as a result of modifications to the crystalline morphology and interactions between the polymer and the filler^71^. The altered internal structure may reduce the energy required to begin melting, causing the crystalline regions to become thermally active at slightly lower temperatures. Nanocomposites follow a similar trend in ${T}_{onset}$, though the shift is slightly less pronounced. From 151 °C (B0), the ${T}_{onset}$ decreases to 144 °C across nanofiller concentrations (N5–N20). This drop is likely due to the higher surface area and better dispersion of nanofillers, which interact more effectively with the polymer chains, slightly disturbing crystalline ordering. Thus, while both filler types reduce ${T}_{onset}$, the mechanisms may differ in detail, with nanofillers promoting finer-scale structural disturbances.

In contrast, the ${T}_{m}$ values exhibit an increasing trend in both bulk and nanocomposite systems, reflecting enhanced crystalline stability. For bulk composites, ${T}_{m}$ increases from 162 °C (B0) to 166 °C for B15 and B20. This could be because the filler functions as a nucleating agent, which encourages crystal perfection^70^. Nanocomposites show an even greater increase, with ${T}_{m}$ reaching 167 °C for N20, the highest among all samples. This supports the idea that nanofillers are more effective nucleating agents due to their large surface area and strong interactions with the polymer matrix. Their ability to refine the crystalline structure leads to higher thermal stability in the melting phase.

Finally, the ${T}_{Endset}$ increases in both systems, further reflecting improved crystalline thermal endurance. For bulk composites, ${T}_{Endset}$ rises from 171 °C (B0) to 178 °C (B20), suggesting that the presence of fillers stabilizes the later stages of melting. In nanocomposites, the trend is more pronounced, with ${T}_{Endset}$ increasing up to 180 °C (N20). This indicates a broader melting range and enhanced thermal resistance of crystalline domains in nanofilled PP. This also suggests the crystalline domains in the nanocomposites that have improved the thermal endurance. In summary, while both bulk and nanofillers significantly improve PP’s thermal properties, nanocomposites show more notable improvements in melting characteristics, making them more suitable for applications that demand greater thermal stability.

**Fig. 21:**
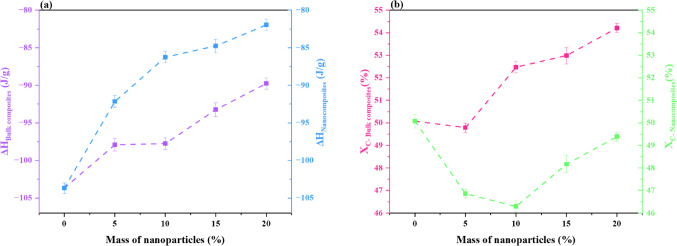
(**a**) Enthalpy of Fusion ($\Delta H$) and (**b**) Degree of Crystallinity $({X}_{C})$ against the Mass of Nanoparticles.

Further information about the crystallinity and thermal behavior of PP composites with different concentrations of bulk and nano-sized fillers is shown in Fig. 21. The heat of fusion was calculated by the integration of the endothermal peak area in the evaluated temperature range.

3 $${X}_{C} (\%)= \frac{\Delta H}{\left(1-{W}_{a}\right){\Delta H}_{0}}\times 100,$$

where ${W}_{a}$ a is the actual mass fraction of bulk and nanofiller, ${\Delta H}_{0}$ is the heat of fusion of 100% crystalline polypropylene (${\Delta H}_{0}$= 207 J⋅g^-1^^73^), ${X}_{C}$ is the crystallinity of the semi-crystalline polymer, and $\Delta H$ is the heat of fusion of the semi-crystalline polymer and its composites.

For all systems, as seen in Fig. 21 (a), $\Delta H$ rose with filler concentration; however, the increase was more pronounced in nanocomposites. From ~ -105 J/g in the PP (B0) increases to ~ -90 J/g in B20 and -83 J/g in N20. The effective nucleation brought about by evenly distributed nanoparticles is probably the cause of this increase in crystal content and/or crystal perfection. At greater loadings, however, the plateauing of $\Delta H$ indicates either a saturation of the nucleating effect or possible agglomeration impeding future advancements^74^. Figure 21(b) illustrates this improved nucleation, with ${X}_{C}$ for bulk composites, increasing gradually from B5 to B20 and peaking at about 54%, demonstrating a pronounced reinforcing effect. On the other hand, nanocomposites show a notable rebound in ${X}_{C}$ at N15 and N20 after initially declining at low filler concentrations (N5 and N10), most likely because of nanoparticle agglomeration or interference with chain mobility. Higher concentrations cause a rise in crystallinity that approaches or equals that of bulk composites due to the enhanced dispersion and matrix interaction of nanoparticles^74^. Partial nanoparticle agglomeration and a lack of nucleation sites can impair polymer chain organization at low filler loadings (N5/N10), which can result in a minor decrease in overall crystallinity. Better dispersion and more nanoparticles give more efficient nucleation sites at higher loadings (N15/N20), which encourage crystallization and raise the degree of crystallinity. This pattern is in line with research on PP nanocomposites, which shows that while an ideal filler content increases nucleation efficiency, a low filler content may cause aggregation consequences^64^. These results demonstrate that although filler addition is advantageous for both types of composites, nanocomposites exhibit greater promise when evenly distributed, but they need to be carefully optimized to prevent initial suppression of crystallinity. Metal oxide fillers are nucleating agents, as shown by the rise in melting temperature and crystallinity seen in Fig. 20 and Table 4. This conclusion is corroborated by XRD data, which reveal that composites containing nanofillers exhibit sharper and more intense diffraction peaks than those containing bulk fillers. Because of their improved dispersion and larger surface area, which provide more nucleation sites for PP crystallization, nanoparticles have a stronger effect.

**Table 4:** DSC results calculated for PP polymer composites with bulk and nanocomposites.

Sample	DSC Results		
	${T}_{onset}$(°C)	${T}_{m}$(°C)	T_*Endset*_(°C)
B0	151	162	171
B5	148	164	175
B10	148	165	177
B15	145	166	177
B20	144	166	178
N5	147	164	178
N10	146	165	178
N15	145	166	179
N20	144	168	180

## Conclusion

This study effectively illustrates how mechanical milling affects the morphological, optical, vibrational, and structural characteristics of Bi_2_O_3_–CuO composites in addition to their function as fillers in PP matrices. XRD and FTIR measurements showed that the milling technique introduced microstrain and drastically reduced crystallite sizes while maintaining the monoclinic crystal phases of both oxides. Indicating tunable optical behavior, photoluminescence studies revealed higher defect-related emissions brought on by oxygen vacancies created by prolonged milling. With EDX verifying elemental purity, morphological analyses verified the change from bulk agglomerates to more homogeneous nanoparticles. The addition of bulk and nano Bi_2_O_3_–CuO fillers to PP significantly improved its thermal performance. As the filler content increased, TGA investigations showed greater thermal stability. Because of their superior dispersion and interfacial interaction, nanocomposites outperformed their bulk counterparts. Higher activation energies and better-controlled degradation behavior provided greater evidence for this. Furthermore, DSC analysis showed better polymer-filler compatibility, specifically in nanocomposites, with increased crystallinity and enthalpy of fusion. The results indicate that Bi_2_O_3_–CuO nanofillers are promising candidates for use in high-performance polymer systems and optoelectronics because they not only improve the optical and structural characteristics of the oxide system but also greatly increase the thermal stability of PP-based composites.

## Data Availability

The datasets used and/or analyzed during the current study are available from the corresponding author on reasonable request.
